# Changing fluxes of carbon and other solutes from the Mekong River

**DOI:** 10.1038/srep16005

**Published:** 2015-11-02

**Authors:** Siyue Li, Richard T. Bush

**Affiliations:** 1 Southern Cross GeoScience, Southern Cross University, New South Wales, 2480, Australia; 2 Key Laboratory of Reservoir Aquatic Environment, Chongqing Institute of Green and Intelligent Technology, Chinese Academy of Sciences, Chongqing 400714, China

## Abstract

Rivers are an important aquatic conduit that connects terrestrial sources of dissolved inorganic carbon (DIC) and other elements with oceanic reservoirs. The Mekong River, one of the world’s largest rivers, is firstly examined to explore inter-annual fluxes of dissolved and particulate constituents during 1923–2011 and their associated natural or anthropogenic controls. Over this period, inter-annual fluxes of dissolved and particulate constituents decrease, while anthropogenic activities have doubled the relative abundance of SO_4_^2−^, Cl^−^ and Na^+^. The estimated fluxes of solutes from the Mekong decrease as follows (Mt/y): TDS (40.4) > HCO_3_^−^ (23.4) > Ca^2+^ (6.4) > SO_4_^2−^ (3.8) > Cl^−^ (1.74)~Na^+^ (1.7) ~ Si (1.67) > Mg^2+^ (1.2) > K^+ (^0.5). The runoff, land cover and lithological composition significantly contribute to dissolved and particulate yields globally. HCO_3_^−^ and TDS yields are readily predicted by runoff and percent of carbonate, while TSS yield by runoff and population density. The Himalayan Rivers, including the Mekong, are a disproportionally high contributor to global riverine carbon and other solute budgets, and are of course underlined. The estimated global riverine HCO_3_^−^ flux (Himalayan Rivers included) is 34014 × 10^9 ^mol/y (0.41 Pg C/y), 3915 Mt/y for solute load, including HCO_3_^−^, and 13553 Mt/y for TSS. Thereby this study illustrates the importance of riverine solute delivery in global carbon cycling.

Rivers, particularly large rivers, have a crucial role in the transport and transformation of carbon input to the ocean. Dissolved and solid materials transport by rivers is a key component of regional and global biogeochemical cycles, such as riverine bicarbonate (HCO_3_^−^) which mainly derives from mineral weathering with carbonic acid, a globally important sink for atmospheric CO_2_^1^,^2^,^3^. The annual carbon flux from rivers to the oceans is estimated to be ~1 Pg C/y (0.8–1.2 Pg C/y), of which 0.38 Pg C/y is dissolved inorganic carbon (DIC), 0.17 Pg C/y is particulate inorganic carbon (PIC), and 0.45 Pg C/yr is organic carbon (0.25 Pg C/yr is the dissolved form and 0.2 Pg C/y is the particulate form)^4^. Nevertheless, global estimates of riverine carbon flux to the ocean still suffer from severe data limitations and poor spatial distribution. This global, patchy coverage (i.e., random sampling) and poorly constrained hydrology, results in flux estimates varying from 0.33 Pg C/y reported by Ludwig *et al.*^5^, to much higher rates 0.41 Pg C/y^6^ or 0.45 Pg C/y^7^.

Anthropogenic activities such as land use changes, soil erosion, construction of reservoirs, water extraction for irrigation and energy generation, and human-induced climate change have greatly modified fluxes of elements laterally and vertically along the continuum of the spectrum of land-ocean-atmosphere systems^7^,^8^. The anthropogenic perturbation of solute and particulate matter exports (i.e., HCO_3_^−^, other major ions and total suspended solid (TSS)) through aquatic continuum, potentially has a major effect on global biogeochemical cycling, yet, the extent of human impacts on riverine carbon fluxes remain poorly resolved^7^,^9^. For example, many studies have exmained riverine concentrations and fluxes using opportunistic, one-off field campaigns^1^,^2^,^4^. Data of water chemistry in 1970s–1980s are widely utilised in these types of studies for developing contemporary global, element cycling models^1^,^4^,^9^,^10^. Some recent work has examined riverine solute fluxes from the Mississippi using a comprehensive data-set and systematic approach^3^, to assess the magnitude of anthropogenically enhanced solute fluxes.

Quantifying the historical riverine delivery of dissolved and solid materials provides important insights on the mechanisms driving global element cycles and ongoing anthropogenic changes. The Mekong River has the world’s 8th largest water discharge (ca. 470 km^3^/y), 12th largest length (4800 km) and 21st largest drainage area (795,000 km^2^). It carries tremendous amounts of dissolved and particulate materials, for example, 123 Mt/y of total suspended sediment (TSS) (10th largest in the world) and 41 Mt/y of solute load to the South China Sea^11^. In comparison to TSS flux, there is very little information on solute characterization^12^,^13^,^14^,^15^. Meybeck and Carbonnel^16^ appear to be the first to report solute flux in the Mekong, and their outdated data (1961–1962) have been widely cited in global modelling analyses^1^,^4^,^5^,^17^.

The primary chemical weathering processes in the Mekong River have been deduced from major ions^11^. Yet the estimates of riverine solute flux are constrained by considerable uncertainty, for example with estimates ranging from 41 Mt/y^11^, to 60 Mt/y^16^, to123 Mt/y^1^. The historical changes of water chemistry and carbon export in relation to human impacts are largely unknown for the Mekong River. There remains a significant gap in our understanding of the element cycles in the Mekong, and what it may mean on a local and global scale.

Like other major rivers in Asia, the Mekong is subject to increasing stresses from population growth, dam construction, and intensive agricultural expansion especially in the recent two decades^12^,^18^. We thus hypothesize that such rapid development is likely to shift the historical trends of solute composition (including carbon) and fluxes toward a relative increase in Cl^−^ and SO_4_^2−^ as anthropogenic markers, and overall net decrease in riverine exports of dissolved species due to decreased water flow. A rich dataset spanning almost 9 decades (i.e. 1923–2011), are investigated systematically with a focus on bicarbonate to determine the long-term dynamics of solute flux. We aim to link the global riverine delivery of solute species with controls on riverine DIC exports. Our research makes an original contribution to (1) revealing the historical dynamics of dissolved species flux using an in-depth compilation and analysis of huge data sets, (2) combining data on the Mekong River and other large rivers to gain insights into how land use, hydrology and lithology control riverine HCO_3_^−^ and other solutes globally and (3) scaling to re-visit estimates of global riverine carbon export.

## Results

### Water discharge and TSS load

**Figure 1:**
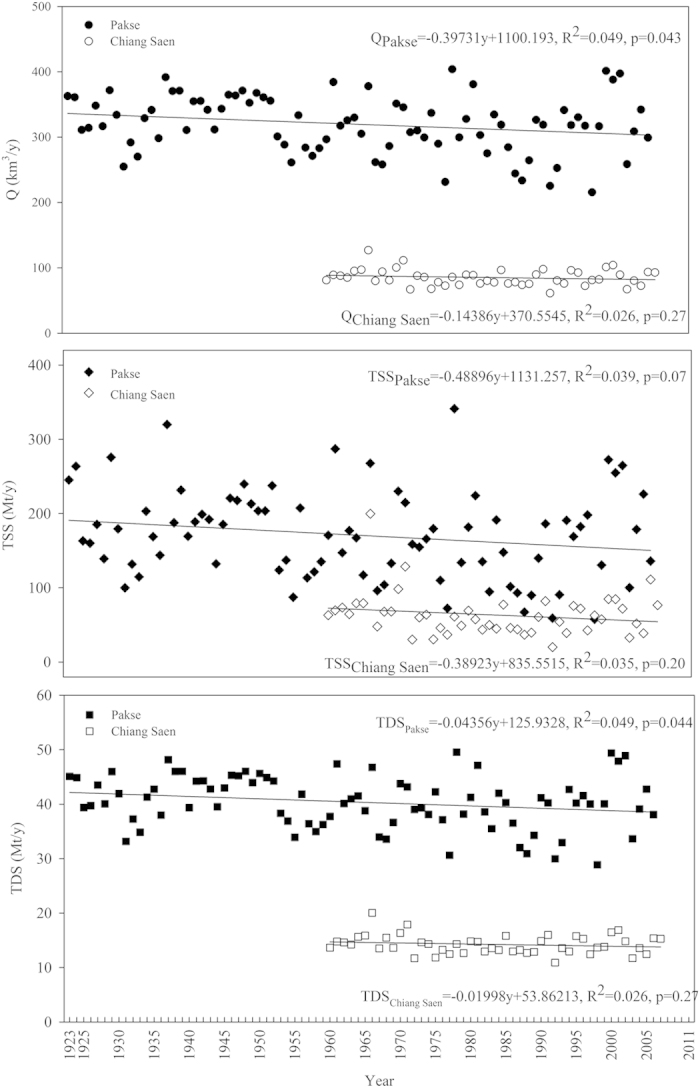
Historical changes of water discharge (**a**), TSS (**b**) and TDS (**c**) fluxes at Chiang saen and Pakse stations of the Lower Mekong River.

The multiyear averaged (1923–2006) water discharge to the sea, as measured at Pakse, the farthest downstream station, is 320 km^3^/y, 3.75-fold the discharge at the upper stream, Chiang Saen station over 1960–2007 (Table S1). Annual water discharge at Chiang Saen ranged from 61 km^3^/y (1992) to 127 km^3^/y (1966) with hydrological extremes in 1966, 1971 and 2001 (flood years), and 1972, 1992 and 2003 (drought years) (Fig. 1a). Annual discharge at Pakse shows the hydrological extremes: major floods in 1978, 2000 and 2002; severe drought in 1977, 1992 and 1998 (Fig. 1a).

The annual TSS load aligned with water discharge at both stations (R^2^ = 0.92, p < 0.01). The River carries a TSS load of 171 Mt/y, 2.7-fold the load from the upper River (Table S1). Annual variations of TSS loads (20–200 Mt/y at Chiang Saen, and 58–341 Mt/y at Pakse) were more variable than annual water discharges (Fig. 1b). Both variables, however, slightly decreased with year (statistically significant at Pakse; p < 0.05).

The contributions of water discharge and TSS load from the upper River to Pakse were respectively 20.1% (1975) −37.7% (1998), and 17.1% (1975) −108.8% (1998) with the extremes coinciding with both annual discharge and TSS load at Pakse (Fig. S5). Overall, 29% of the variance of discharge in the lower Mekong could be explained from the upper River, while only 25% of TSS load could be explained which was lower than expected, based on the proportion (i.e., 35%) of total drainage area (reflected by regression analyses; Figs S6a and S6b).

### HCO_3_^−^ and other major ion concentrations

**Figure 2:**
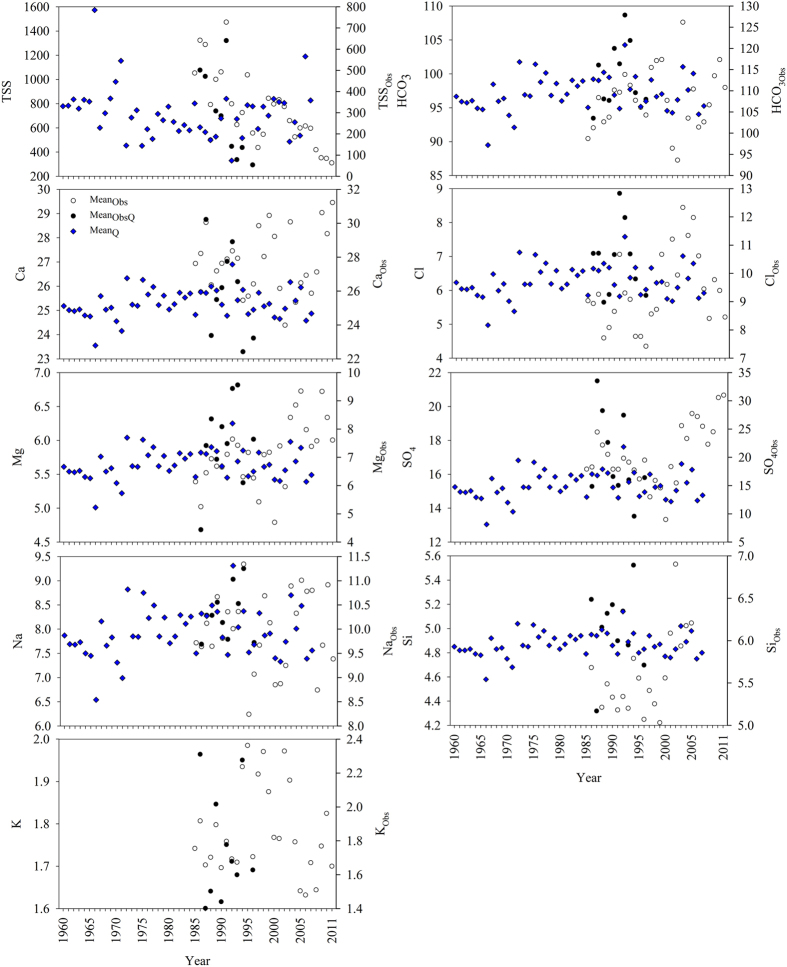
Historical changes of major ion concentrations at Chiang Saen station, Mekong River (unit in mg/L) (Note: no predictable annual K^+^ is due to its weak relationship to flow volume) (Mean_Obs_ is measured concentrations, Mean_ObsQ_ is measured discharge-weighted concentrations, Mean_Q_ is discharge-weighted concentrations from models).

**Figure 3:**
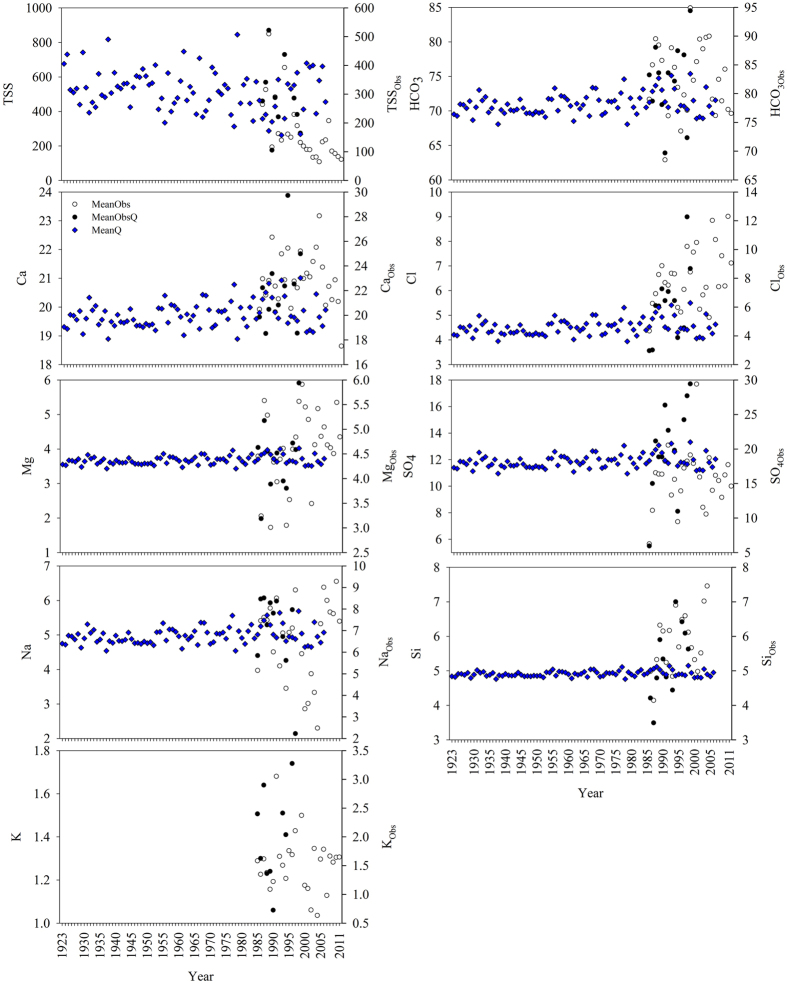
Historical changes of major ion concentrations at Pakse station, Mekong River (unit in mg/L) (Note: no predictable annual K^+^ is due to its weak relationship to flow volume) (symbols see Fig. 2).

The annual flow-weighted concentrations of HCO_3_^−^ and other major ions in the LMR are shown in Figs 2 and 3, and supporting statistical details in Table S2. The total cationic charge (Tz^+^ = Na^+^ + K^+^ + 2 Ca^2+^ + 2 Mg^2+^, unit in μmol_c_/L) (mol_c_ denotes moles of charges) ranged between 1644 and 1979 μmol_c_/L with an average of 1858 μmol_c_/L at Pakse, 75% the average at Chiang Saen (Table S2, date from Figs 2 and 3). The total anionic charge (Tz^−^) ranged from 1548 to 2310 μmol_c_/L with an average of 1909 μmol_c_/L. The flow-weighted Tz^+^ at the lower station was 1548 μmol_c_/L (range: 1465–1671 μmol_c_/L), 1.4 times the average for the world’s rivers (1123 μmol_c_/L), while lower than most of the Himalayan rivers (Table S4). The flow-weighted Tz^−^ ranged from 1455 to 1668 μmol_c_/L with an average of 1539 μmol_c_/L. Thus, the normalized ionic charge balance (NICB = (Tz^+^−Tz^−^)/Tz^+^) were well balanced.

The annual TDS concentration showed significant spatial variability between stations (P < 0.01 by ANOVA), with values ranging between 167 and 221 mg/L at Chiang Saen, and between 132 and 164 mg/L at Pakse, respectively. Annual concentration of TDS was, on average, 1.3 times higher at Chiang Saen than at Pakse (189 vs 149 mg/L respectively). The multiyear flow-weighted mean of TDS (122 mg/L) at Pakse was slightly higher than world discharge-weighted average (97 mg/L) (Table S4).

Concentrations of major elements (except for Si) showed significant annual differences between Chiang Saen and Pakse stations (P < 0.01 by ANOVA). Na^+^, K^+^, Cl^−^ and SO_4_^2−^, were more variable at Pakse, while HCO_3_^−^ and Ca^2+^ were least variable at both stations. Similar to most of the Himalayan Rivers, water composition was dominated by Ca^2+^ and HCO_3_^−^, comprising 71% of the total TDS load at Pakse station. Calcium was the most abundant cation with annual concentrations of 17.5–28.1 mg/L and followed by sodium (2.5–9.3 mg/L). Potassium was the least abundant major cation with an annual content of 0.6–3.1 mg/L. Annual flow-weighted means of major cations decreased as follows (unit in mg/L): Ca^2+^ (19.7) > Na^+^ (5.0) > Mg^2+^ (3.7) > K^+^ (1.5). HCO_3_^−^, the most abundant major anion, varied from 68.5 to 95.0 mg/L. The second most abundant major anion was SO_4_^2−^ ranging from 6.3 to 29.4 mg/L. Annual flow-weighted means of major anions were, in descending order (unit in mg/L): HCO_3_^−^ (71.0) > SO_4_^2−^ (11.9) > Si (4.9) ~ Cl^−^ (4.5) (Fig. 3; Table S2M). Two-thirds of the annual cation load was made up of Ca^2+^; HCO_3_^−^ made up 76% of the anions (Tables S2 and S3). However, Chiang Saen station showed a different flow-weighted trend of major anions in the following order (unit in mg/L): HCO_3_^−^ (97.2) > SO_4_^2−^ (15.4) > Cl^−^ (6.3) > Si (4.9) (see Table S2).

Ternary plots shown in Fig. S7, show samples cluster close to Ca^2+^ in the cation plot, and to the HCO_3_^−^ apex in the anion plot. Ca^2+^ contributed 30–75% (mean: 59.4%) to the major-cation budget at Chiang Saen station, its contribution increased to 40–90% at Pakse. HCO_3_^−^ accounted for 60–90% of the major anions at Chiang Saen, its contribution to the major-anion budget at Pakse was 50–85%.

### HCO_3_^−^ and other solute fluxes

**Figure 4:**
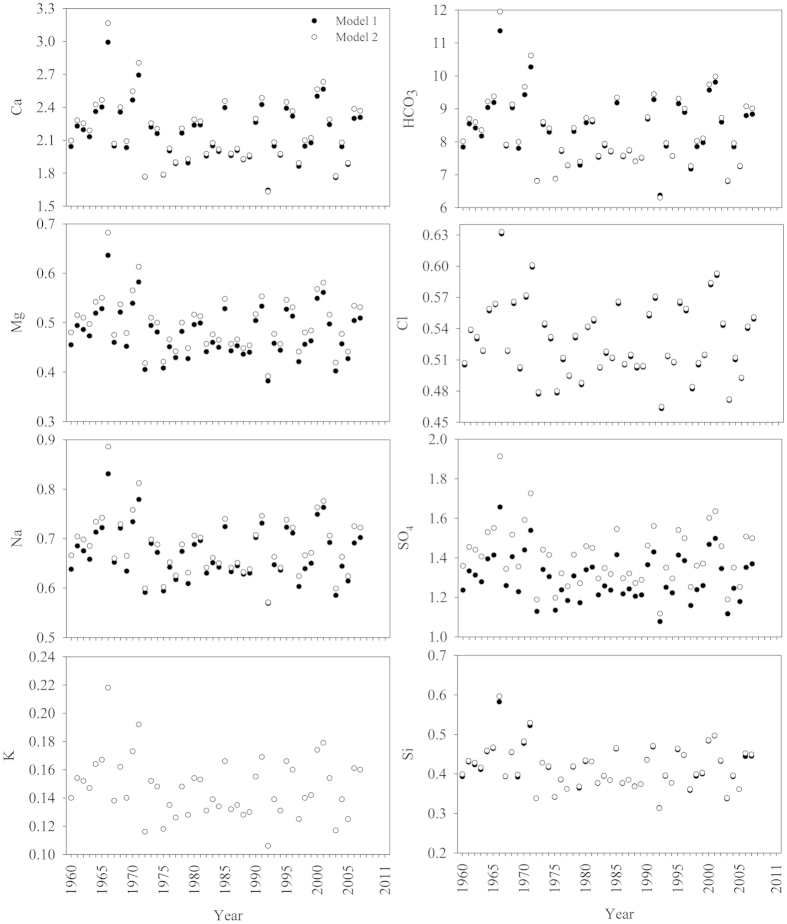
Historical fluxes of major ions at Chiang Saen station, Mekong River (unit in Mt/y).

**Figure 5:**
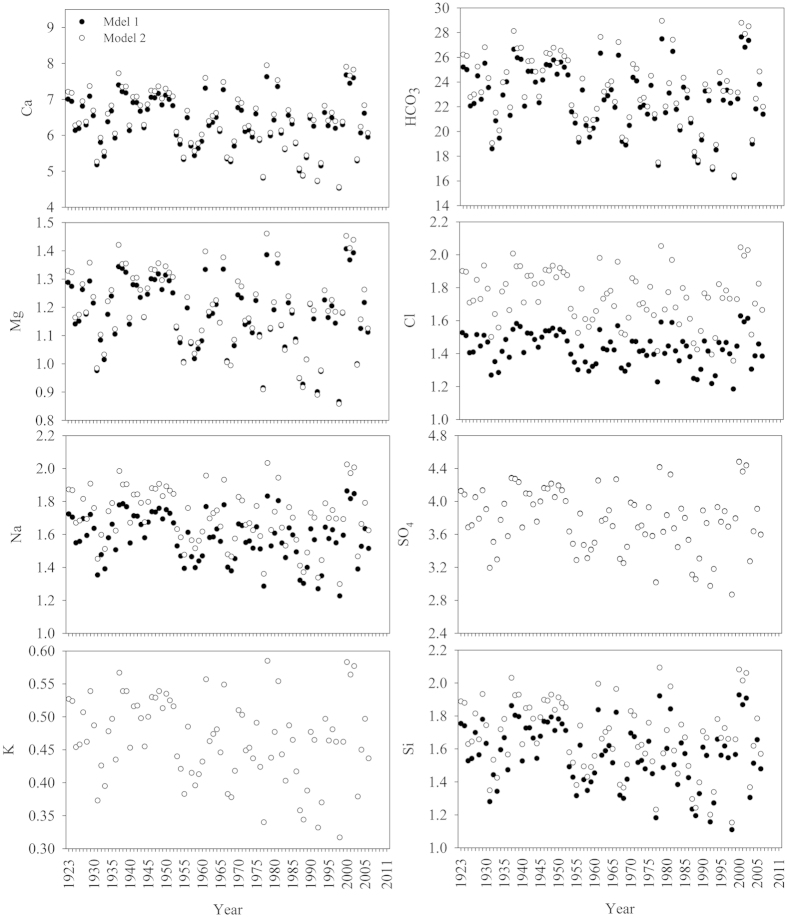
Historical fluxes of major ions at Pakse station, Mekong River (unit in Mt/y) (statistical decreasing fluxes of bicarbonate and other solute species with year, particularly remarkable decreases in bicarbonate and Ca^2+^ fluxes in the recent decade).

Annual fluxes of HCO_3_^−^ and other solutes had universal patterns with annual water discharge (R^2 ^> 0.99, p < 0.001), resulting in that annual fluxes of HCO_3_^−^, other major ions and TDS peaked in the year with highest flow, and reached the minimums in the year with lowest discharge (Figs 1,4 and 5). Fluxes of TDS and major ions had comparable variation factors with water discharge at both stations (i.e., 1.3–1.9 for major ion fluxes, while 2.1 for water discharge at Chiang Saen; 1.4–1.8 for major ion fluxes, and 1.9 for water discharge at Pakse). In upper Chiang Saen station, the lowest water discharge in 1992 accordingly resulted in the lowest fluxes of solute species: TDS (10.9 Mt/y), Ca^2+^ (1.6 Mt/y), Na^+^ (0.6 Mt/y), Mg^2+^ (0.4 Mt/y), K^+^ (0.1 Mt/y), HCO_3_^−^ (6.3 Mt/y), SO_4_^2−^ (1.1 Mt/y), Cl^−^ (0.5 Mt/y) and Si (0.3 Mt/y). Their highest fluxes were recorded in the peak-flow year of 1966 as follows: TDS (20 Mt/y), Ca^2+^ (3.2 Mt/y), Na^+^ (0.9 Mt/y), Mg^2+^ (0.7 Mt/y), K^+^ (0.2 Mt/y), HCO_3_^−^ (12 Mt/y), SO_4_^2−^ (1.9 Mt/y), Cl^−^ (0.6 Mt/y) and Si (0.6 Mt/y) (Figs 1 and 4). At Pakse station, annual fluxes were respectively 28.9–49.6 Mt/y for TDS, 4.6–8 Mt/y for Ca^2+^, 1.3–2 Mt/y for Na^+^, 0.9–1.5 Mt/y for Mg^2+^, 0.3–0.6 Mt/y for K^+^, 16.5–29 Mt/y for HCO_3_^−^, 2.9–4.5 Mt/y for SO_4_^2−^, 1.4–2.1 Mt/y for Cl^−^ and 1.2–2.1 Mt/y for Si. Their lowest fluxes consistently occurred in 1998 and highest in 1978 (Figs 1 and 5).

Spatial characterization of solute fluxes and yields are shown in Table S3. The annual fluxes were significantly higher at Pakse than at Chiang Saen (p < 0.01 by ANOVA), the areal yields, however, were the same between the two stations. Contributions (34.1–35.8%) of multiyear averaged fluxes of TDS, and dominant ions (i.e., HCO_3_^−^ and Ca^2+^) from the upper River to Pakse were similar to the contribution of drainage area (34.7%). The contributions of all the solute constituents (Si excluded) from the upper River to Pakse were higher than the contribution of water discharge (26.7%). Totally, solute fluxes in the LMR were, in descending order (Mt/yr): TDS (40.4) > HCO_3_^−^ (23.4) > Ca^2+^ (6.4) > SO_4_^2−^ (3.8) > Cl^−^ (1.74) ~ Na^+^ (1.7) ~ Si (1.67) > Mg^2+^ (1.2) > K^+^ (0.5).

### Long-term trends

Annual concentrations of TSS significantly decreased at both stations, while Si and anthropogenic markers (Cl^−^ and SO_4_^2−^) at Chiang Saen, and Cl^−^ and possibly Si at Pakse significantly increased (Figs 2 and 3; Table S5). The modelled, discharge-weighted concentrations of major elements slightly, but statistically significantly, increased at the lower station, however, a significant decrease in annual water discharge resulted in a statistically decreasing flux of TDS and major ions (SO_4_^2−^ excluded) in the Mekong. With regard to annual concentrations in the recent decade (2001–2011), significant increases with higher coefficients in Na^+^, Cl^−^ and Si (R^2 ^> 0.43, p < 0.05 by Spearman’s rho), while significant decreases in Ca^2+^ and HCO_3_^−^ (R^2 ^> 0.49, p < 0.05 by Spearman’s rho) were observed at the lower Pakse station. Our analyses corroborated the previous hypothesis of persistent increase in Cl^−^ and SO_4_^2−^ concentration^11^.

**Figure 6:**
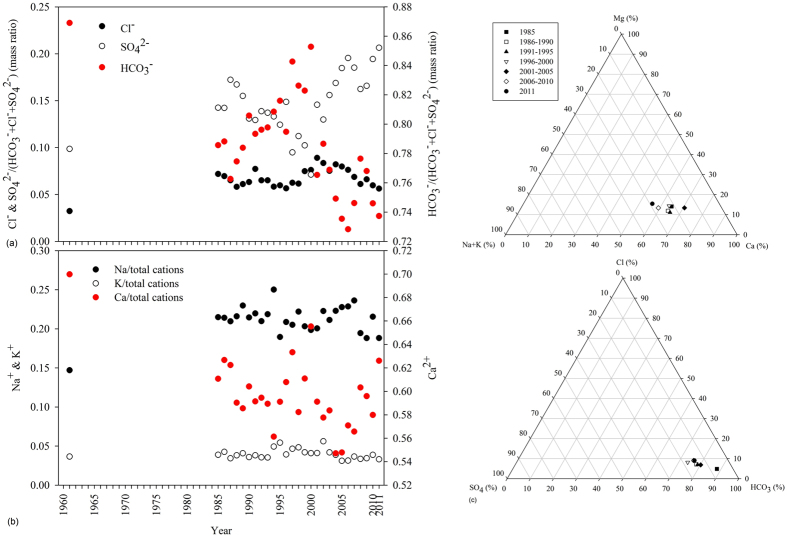
Evolution of relative importance of HCO_3_^−^, SO_4_^2−^ and Cl^−^ (**a**), and the percentage content of Na^+^, K^+^ and Ca^2+^ in the sum of cations (**b**) with time at Chiang Saen, as well as historical changes of the relative dominance of major ions at Pakse (**c**), Mekong River.

The rate of increase (ROI) of specific ions was markedly different: 0.06 mg/L/y for Cl^−^ and 0.33 mg/L/y for SO_4_^2−^ at Chiang Saen; 0.38 mg/L/y for Cl^−^, and 0.54 mg/L/y for Na^+^ in the recent decade at Pakse. Compared with 50 years ago, the abundance of Cl^−^ and SO_4_^2−^ at Chiang Saen have changed greatly, increasing from 3.2% to 6.7% for Cl^−^, and from 9.9% to 20.6% for SO_4_^2−^ (Fig. 6a). The relative abundance of Na^+^ has also increased from 14.7% to 21.1%, while the relative abundance of Ca^2+^ and HCO_3_^−^ has gradually decreased (Fig. 6a,b). At the lower Pakse station, the consistent increase of Cl^−^ has enhanced its relative abundance by a factor of 2, and the relative abundance of Na^+^ + K^+^ accordingly increased by a factor of 1.8 (Fig. 6c).

### Comparison of the Mekong to other large rivers

The data from a comprehensive analysis of 44 of the world’s major rivers are presented in Table S4a–4e. HCO_3_^−^ concentration in the Mekong is within the intermediate range relative to other large rivers. For example, HCO_3_^−^ concentration is comparable to other large Asian monsoon rivers such as Brahmaputra and Indus, and Ob. HCO_3_^−^ concentration is highest (>3000 μM) in the Danube, Don, Salween and Yellow, while Orinoco, Indigirka, Zaire, Yana and Amazon have very low HCO_3_^−^ concentrations (<350 μM). A similar spatial pattern is observed for TDS concentrations (reflected by r = 0.82, p < 0.01); Don has the highest content (>800 mg/L), followed by Yellow, Murray and Danube (>430 mg/L). TDS concentration in the Mekong is much lower than that of most Himalayan rivers (Yangtze, Yellow, Irrawaddy, Salween), but 4.5-fold that in Orinoco, 4-fold that in Yana and Zaire, and 3.2-fold that in Amazon and Indigirka, and these rivers have the lowest TDS concentrations in the world. Both concentrations of TDS and HCO_3_^−^ in the Mekong are 1.4 times higher than global water-discharge weighted average.

Specific fluxes of HCO_3_^−^ (681 × 10^3^ mol/km^2^/y), TDS (74.1 t/km^2^/y) and TSS (313.2 t/km^2^/y) delivered by the Mekong are considerably higher than the global averages by a factor of greater than 3 (3 for TDS, 3.2 for HCO_3_^−^ and 3.7 for TSS). Particulate and dissolved specific flux in Asian monsoon rivers, particularly in the Himalayan Rivers, are remarkably higher than other continental rivers such as Zaire, Zambezi, Senegal especially Eurasian rivers (i.e., Indigirka, Kolyma and Yana) in the high latitude zone. For example, HCO_3_^−^ specific flux is highest in Himalayan Rivers such as Irrawaddy (2257 × 10^3^ mol/km^2^/y), Salween (2256 × 10^3^ mol/km^2^/y) and Red (2017 × 10^3 ^mol/km^2^/y). The moderate HCO_3_^−^ specific flux in the Mekong among the Himalayan Rivers is around 100 times higher than that of Senegal (7 × 10^3^ mol/km^2^/y). TDS specific flux in the Mekong is 20-fold higher than that of Yana (3.6 t/km^2^/y), and 14-fold higher than that of Indigirka (5.4 t/km^2^/y). The ratio could be as high as 322 for HCO_3_^−^ and 64 for TDS when Irrawaddy is considered. Areal yield of HCO_3_^−^ accounts for 52% (range: 21.4%–83.5%) of TDS yield and they show strong couplings at global scale (r = 0.97, p < 0.01). Thus, both HCO_3_^−^ and TDS specific fluxes in the Mekong are comparable to Ganges, Brahmaputra and St. Lawrence. The TSS specific flux peaks in the Yellow (1184 t/km^2^/y), which is around 237 times higher than the Lena, and 3.8-fold higher than the Mekong.

HCO_3_^−^ flux (371 × 10^9^ mol/y) to the sea by the Mekong (measured at Pakse) is 18% of the Amazon, 19% of the Yangtze, 3.5 times of the Yellow, slightly lower than Brahmaputra, Danube, Mackenzie, Parana and Xijiang, while similar to Lena, Nile and Tigris. The dissolved flux (40.4 Mt/y) exported by the Mekong is 17% of Amazon, 22% of Yangtze, 2.5 times of the Yellow, slightly lower than Brahmaputra, Danube, Mackenzie, Ob, Parana, Salween and St. Lawrence, but very similar to Mackenzie, Nile, Xijiang, Tigris and Zaire. The Mekong (above Pakse station), comprising 0.37% of the global continental area, supplies 1.16% of riverine HCO_3_^−^ flux, 1.12% of TDS and 1.35% of TSS in the world, slightly higher than its contribution (0.85%) to global river discharge.

### Global relations between solutes and land use, hydrology and lithology

**Figure 7:**
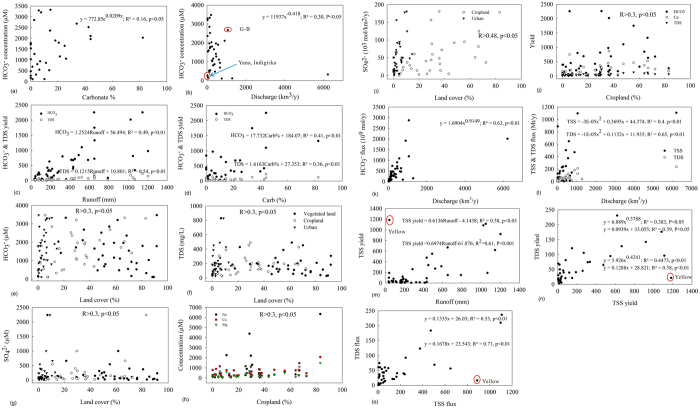
Relationships between dissolved and particulate species and basin physical characteristics (lithology, hydrology, land cover and human activities) ((**b**)-regression model after deletion of three rivers, G-B is Ganges-Brahmaputra (**e–g**) have same symbols; (**m–o**)-regression models below do not include Yellow River) (yields and fluxes of TDS and TSS are respectively expressed in Mt/y and t/km^2^/y; yields and fluxes of ions are respectively expressed in 10^9 ^mol/y and 10^3 ^mol/km^2^/y).

River HCO_3_^−^ concentration is significantly correlated with the percentage of carbonate mineral composition (Carb%) (R^2 ^= 0.16, p < 0.05; Fig. 7a). On a global scale the correlation between river HCO_3_^−^ concentration and water discharge is complex and not strong, and correlations are significantly negative when three rivers are excluded (R^2 ^= 0.3, p < 0.05; Fig. 7b), consistent with the relationships for individual rivers (Fig. S2 and S3). In contrast to the concentration of dissolved species, specific fluxes of all the particulate and dissolved variables (TSS, HCO_3_^−^, other major elements and TDS) are positively related to runoff (water discharge per unit area, unit in mm) (Fig. 7c; yields of TDS and HCO_3_^−^ *vs* runoff are shown). The percent of carbonate is significantly related to the areal yield of HCO_3_^−^, K^+^, Ca^2+^, Mg^2+^ and TDS (Fig. 7d; yields of TDS and HCO_3_^−^ *vs* Carb% are shown). By using multiple regression to target HCO_3_ flux with regard to Carb% and runoff, the r^2^ value increases to 0.69 (HCO_3_ yield (10^3 ^mol/km^2^/y) = −27.593 + 0.817 × runoff (mm) + 11.894 × Carb%; R^2 ^= 0.69, p < 0.001).

Landscape factors such as land cover and human activities also contribute to concentrations and yields of HCO_3_^−^ and other solutes. Forested and vegetated lands have low major ions, while anthropogenic activities such as cropping and urbanization positively contribute to dissolved solutes, particularly SO_4_^2−^ (Fig. 7e–h). Grass is negatively related to yields of dominant ions Ca^2+^ and HCO_3_^−^, as well as SO_4_^2−^; SO_4_^2−^ yield is also significantly related to vegetated land. The yields of the three variables (Ca^2+^, HCO_3_^−^ and SO_4_^2−^), however, are positively correlated with cropland, and urban land -use is exclusively and significantly related to SO_4_^2−^ yield (Fig. 7i,j). Surprisingly, population density appears weakly correlated to major ion concentration except for HCO_3_^−^, while it is significantly related to yields of HCO_3_^−^, TSS and Mg^2+^.

Riverine fluxes of HCO_3_^−^, other solute species and TSS are as expected, largely controlled by the magnitude of river discharge (Fig. 7k,l). Other factors such as basin area, runoff and Carb% are also important, particularly for DIC. Similar to individual rivers (Figs S2 and S3), the flux of HCO_3_^−^, TDS and TSS can be well predicted using water discharge (R^2^ ranges from 0.4–0.65, p < 0.01; Fig. 7k,l).

## Discussion

### Mechanisms controlling HCO_3_^−^ and other solutes: natural processes

The plot of the TDS concentrations vs the weights of Cl^−^/(Cl^−^ + HCO_3_^−^) and Na^+^/(Na^+^ + Ca^2+^) was characterized by a low-to-moderate TDS concentration and low weight ratios of Cl^−^/(Cl^−^ + HCO_3_^−^) (<0.1) and Na^+^/(Na^+^ + Ca^2+^) (<0.3) (data from Table S2). Further, samples fell in the clusters near Ca^2+^ and HCO_3_^−^ apexes in the ternary plots (Fig. S6). The abundance of carbonate rocks in the drainage catchment and the aforementioned indicators in the aqueous data suggested that the Mekong river waters appear to be dominated by chemical weathering of carbonates. This determines that Mekong’s water chemistry, typically characterized by the Ca^2+^-HCO_3_^−^ type, is consistent with the catchment’s geography.

Mass balance and stoichiometry were used to identify the contributions of various rocks to cations, based on Fig. S8. Based on our analysis, at Chiang Saen, silicates contributed to 21% of cations, while carbonates contributed a total of 79% (52.4% for calcite and 26.6 for dolomite) to cations. This was very different at Pakse, where silicates were 16.5%, and 63.3% for calcite and 20.2% for dolomite. The contribution of silicate was higher than the reported values (0.05–0.2) in the Yangtze^19^, a carbonate typical river with 2 times higher carbonate composition than the Mekong (44% vs 21% for the Yangtze and Mekong)^4^.

### Anthropogenic impacts

Potential large impacts from China’s dams on water flow and thus TSS load in the Lower Mekong at Chiang Saen have been reported, with the Chinese cascade dams at the centre of international debate^12^,^15^. Our data demonstrate that low discharge years of 1992 and 2003 were consistent with the impoundment of Manwang Reservoir in 1992, and Dachashan Reservoir in 2003. A greater frequency of floods in the 1960s–1970s, and a period of persistent droughts in recent years have contributed to a lower post-dam flow (1992–2007; averaging 83.9 km^3^/y) by comparison to the pre-dam period (1960–1991; averaging 85.8 km^3^/y). These data circumstantially support the general opinion that anthropogenic dams in Yunnan (China) have significantly altered the Mekong hydrography. However, there is an alternative conclusion supported by the following evidences: (a) both reservoirs’ combined active storage capacity of 0.63 km^3^ is minor when compared to the total annual water flow at Chiang Saen (85.2 km^3^/y over 1960–2007; Fig. 1a), and (b) the relationship between rainfall in the upper catchment and discharge at Chiang Saen (R^2^ = 0.42; p < 0.01) was maintained, and the years with maximal- and minimal- precipitation were consistent with our observed water discharge trend (Fig. 1; Fig. S9). Further, the ratio of precipitation to runoff (P/R) remains constant (Fig. S9d). Water discharge at Chiang Saen is therefore predominantly dominated by rainfall in the upper catchment, even though this region is experiencing dramatic changes such as human-induced global warming (+3 °C/100 years) (Fig. S9a), dam construction, land use change, and potential increases in domestic and industrial water use.

Similar to the adjacent Asian Rivers such as the Yangtze and Yellow^20^,^21^, human activities are evident in the long-term increasing trend in the concentration of anthropogenic markers (Cl^−^, Na^+^ and SO_4_^2−^). For example, the persistent increase of Cl^−^ and SO_4_^2−^ in the upper Mekong (measured at Chiang Saen), as well as abrupt increases in Cl^−^ and Na^+^ during the recent decade at Pakse (Fig. 3; Table S5). We ascribe the increase of sulphate (0.33 mg/L/y) to acidic deposition in the Yunnan Province of China, while dramatic increases in Cl^−^ and Na^+^ at Pakse (0.38 mg/L/y for Cl^−^ and 0.54 mg/L/y for Na^+^) are particularly due to domestic discharges of salts. This is reasonable, as the Mekong basin is dominated by rural development and relatively little intensive urbanization. The ROIs in the Mekong are consequently much lower than those in the Yellow River (2.8 mg/L/y for Cl^−^ and 2.47 mg/L/y for Na^+^), a typical large Asian river with intensive anthropogenic stressors such as high urbanization and industrialization, and water withdrawal/extractions for extensive agrarian purposes^21^.

Data on δ^34^S-SO_4_^2−^sufficient to resolve sulfur sources, either from sulfide oxidation or from gypsum dissolution in the Mekong River catchment, are not available. However, along the Mekong, δ^18^O-H_2_O ranges from −9%–4% and δD-H_2_O from −27%–52%^22^,^23^. Such evidence, when combined with high stoichiometric ratios of (Ca^2+ ^+ Mg^2+^)/SO_4_^2−^ (4.9 ± 1.4; Mean±S.D.), is considered indirect evidence for minor contributions from gypsum dissolution. This is also corroborated by the adjacent river’s negative δ^34^S-SO_4_^2−^ values from the paleo stratigraphy of abandoned river channels that suggest dominant sources of sulfide oxidation (including coal combustion) for sulfates^24^. These evidences support the human enhanced SO_4_^2−^ concentration in the Mekong and other Asian rivers.

As discussed earlier, the domestic release of salt constitutes an important source of Na^+^ and Cl^−^ in the less-developed precincts along the Mekong River. To demonstrate this we calculate domestic contributions of Cl^−^ based on the population and a conservative per capita human generation rate of 18 kg/capita/y^25^. The current population of 70 million in the Basin thus discharges a Cl^−^ load of 1.26 Mt/y, which accounts for 72.4% of the Cl^−^ delivered by the Mekong. This proportion is comparable to the anthropogenic contribution to Cl^−^ (68.2%) using a forward mass-balance approach^11^.

Prior research also reported large effects of irrigation on major ion chemistry *via* the following processes^21^: (1) Agricultural irrigation results in concentration of major ions because of water consumption of crops and enhanced evapotranspiration. (2) Irrigation water selectively precipitates carbonates in the irrigated lands, leaving ions such as Na^+^, Cl^−^ and possibly SO_4_^2+^, behind in the return water. This process understandably results in selective concentrations of some ions. Irrigation could be a major driver in the Mekong, a river basin with widespread agricultural land. Approximately 40% of the basin is agricultural with more than 100,000 km^2^ of land cultivated for rice production, which results in 42 km^3^/y consumed by irrigated agriculture (the largest water user). Expansion of irrigated agriculture, particularly in the past two decades, has accelerated the selective precipitation of carbonates and subsequent enrichment of Cl^−^ and Na^+^ in the return water, which corresponds very well with the evolving trend of cations and anions in the River (Fig. 6). This process is also partly associated with the decreases in Ca^2+^ and HCO_3_^−^ composition at the lower, Pakse station.

The application of potassium chloride (KCl) in agricultural production is another potentially important contributor to the increase in aquatic Cl^−^ 26. However, the effect of agricultural fertilizer on riverine Cl^−^ is expected to be negligible as (a) no significant trend of K^+^ concentration was observed during the whole period or the recent decade at either station (Figs 2 and 3), and (b) the relative abundance of K^+^ in cations remained consistent (Fig. 6b). Other potential sources for Cl^−^ include road salts used to deice, and atmospheric deposition. The contribution of road salts in the Mekong River can be disregarded due to climate, while atmospheric origin contributes ~12% to Cl^−^ in this area^11^. In total, domestic wastes of salts, irrigation and atmospheric deposition contribute to a 2-fold increase in the relative abundance of Cl^−^ in anions over the past three decades at Pakse (comparison of relative dominance of Cl^−^ in anions during 1985–2011 with the year 1961 at Chiang Saen) (Figs 6a,c).

In contrast to Na^+^ and Cl^−^, Ca^2+^ and HCO_3_^−^ decrease and are closely linked to increasing aquatic photosynthesis. Phytoplankton (e.g., diatoms) assimilates dissolved inorganic carbon and nutrient elements (e.g., N, P, Si) to synthesize organic components based on a specific ratio^27^. Drastic increases in concentrations of nitrate and dissolved inorganic phosphorus (DIP), particularly in the recent decade, in the Mekong River have been reported (i.e., nitrate increased by a factor of 4, and DIP by a factor of 3)^28^. The plentiful N and P loads introduced by human activities and river damming in the drainage basin, understandably boosts aquatic photosynthesis and consequently increase primary production, resulting in a significant decrease of HCO_3_^−^ (possibly Ca^2+^). This trend is in good agreement with that in the Yangtze^29^, our trend, however, is contrary to the Mississippi^3^. The increases in both concentrations and fluxes of bicarbonate are attributable to the increase in discharge from anthropogenic, agricultural watersheds. Regarding to the arid and semiarid Yellow River, historically stable concentration of HCO_3_- results from the combination of high evaporation, fractional crystallization, water withdrawal and enhanced uptake by aquatic organisms. Bicarbonate flux delivered by the River, however, dramatically decreases, owing to the sharp decrease in the seaward water discharge^21^. As discussed above, increasing photosynthesis would increase the biological uptake of dissolve Si by aquatic organisms. As the Si concentration is dependent on the weathering rate of regolith silicates, a net decline in Si would be expected. Our contrary observation, indicates an accelerated chemical and physical weathering rate in the basin^30^,^31^ which can be primarily attributed to human-induced land use change (e.g., deforestation and agricultural expansion).

### Comparative controls on DIC and other global solute characteristics

As mentioned earlier, both hydrology and lithology have a profound effect on HCO_3_^−^ characterization (Fig. 7a–d)^30^,^32^,^33^. Our results further suggest that HCO_3_^−^ concentration is far more strongly related to the precipitation and evaporation balance than lithological composition. For example, Xijiang, has the highest proportion of carbonate minerals (82.4%) and thus higher specific river HCO_3_^−^ flux, while it only has a moderate HCO_3_^−^ concentration due to a high runoff. In contrast, the Don and Yellow have little carbonate in the lithology (5.9% vs 7.6% for Don and Yellow) and thus a low specific HCO_3_^−^ flux. But they have a very high HCO_3_^−^ concentration (>3200 μM) because of much higher evaporation over precipitation (reflected by the ratio of precipitation to runoff, 5.5 for Don and 11 for Yellow). A comparison of the HCO_3_^−^ concentration in the Don and Yellow with that of the Amazon is surprisingly interesting. The carbonate composition in the Don and Yellow is only 1.5–2 times that of the Amazon, while the aqueous HCO_3_^−^ concentration of the former is an order of magnitude higher than the latter, further evidence of the predominant influence of rainfall (1067 mm/y in the Amazon, Table S4; references see supplementary material) over lithology, on carbonate concentration.

Regional and global data suggest that negative δ^34^S-SO_4_^2−^ values in river waters are characteristic of oxidation of sulfide minerals^24^,^31^,^34^, and thus the sulfide-derived SO_4_^2−^ is significantly related to human land use (crop and urban) (Fig. 7g,i); because human land use exacerbates mechanical erosion and thus facilitates continuous exposure of fresh mineral surfaces accelerating the rate of sulfide oxidation. The oxidation of sulfides (including coal combustion) supplies protons (i.e., sulphuric acid) that prompt mineral weathering in river basins. Sulphuric acid from sulfide oxidation, combined with high physical erosion rates from intensive anthropogenic activities, contribute to concentrations and yields of solute species, corroborating our findings of positive associations between solutes and human land use (Fig. 7g–j). Nevertheless, element ratios (Median ± S.D.; unit in moles of charges) such as HCO_3_^−^/(Ca^2+^ + Mg^2+^) (0.84 ± 0.29) and very high Ca^2+^/SO_4_^2−^ (4.69 ± 18.26) for global, large rivers indicate that H_2_SO_4_ does not replace H_2_CO_3_ as a source of protons for rock weathering. Further, most rivers show negative δ^13^C-DIC (i.e., close to or less than −10%)^2^,^28^,^35^, suggesting the prominent source is soil water CO_2_ rather than carbonate. Therefore, HCO_3_^−^ in global rivers mainly comes from rock weathering with carbonate acid and consequently riverine HCO_3_^−^ flux represents a sink of atmospheric CO_2_. However, this carbon sequestration will potentially decline due to human-induced acceleration of sulfide oxidation.

In regard to DIC and other solute fluxes, the four largest river sources of HCO_3_^−^ and TDS to the oceans are the Amazon, Yangtze, Ganges-Brahmaputra and Mississippi, and together they have 17-fold higher fluxes of HCO_3_^−^ and TDS than the Mekong. Thus, the most important four rivers carry a disproportionally high particulate and dissolved material flux, and contribute to 19.4% of the global riverine HCO_3_^−^ flux (31900 × 10^9^ mol/y), and 18.5% of the global solute load (3618.2 Mt/y), this proportion is comparable to the contribution (23.5%) of global river discharge (37,400 km^3^/y), while much higher than the contribution (8.3%) of the world’s continental area (148 M km^2^). The total TSS, TDS and HCO_3_^−^ flux delivered by our selected 44 rivers is respectively 6522  Mt/y, 1884 Mt/y and 16367 × 10^9^ mol/y. The water discharge by these big rivers accounts for 48.1% of the global, total river discharge,. Thus the estimated global HCO_3_^−^ flux would be 34014 × 10^9^ mol/y (0.408 Pg C/y) through upscaling analysis with water discharge. This estimate is somewhat higher than that (0.33 Pg C/y) estimated by Ludwig *et al.*^5^, but proximate to that (0.38–0.41 Pg C/y) cited in Amiotte Suchet *et al.*^4^ (2003) and Mackenzie *et al.*^6^. The calculated global riverine solute load is 3915 Mt/y, representing 108% of the widely cited flux (3618 Mt/y). The estimated global TSS load (13,553 Mt/y) is comparable to the recent update work (12,610 Mt/y), however, much lower than an earlier widely cited number of 20,000 Mt/y^8^.

### Global physical and chemical erosion associations

Several studies have explored the key controls on chemical weathering that include temperature, physical erosion, runoff, lithology, topographical variables (slope and elevation), land cover and tectonic activities^4^,^30^,^35^,^36^,^37^,^38^. The relative role of each of these parameters to chemical erosion is unresolved; here we underscore the importance of runoff, physical erosion and lithological composition.

The comparative assessments of contributions from runoff, lithology, land cover and anthropogenic activities to chemical erosion are shown in Fig. 7c,d,j. Similarly to other rivers^11^,^35^,^36^, runoff is investigated as the primary driver for chemical erosion at the global scale. Yields of TDS (an index of chemical weathering) and TSS (an index of physical erosion) that are commonly used to evaluate kinetic relationships between the rates of chemical and physical erosion (CER and PER) have good positive correlations with runoff (Fig. 7c,m). Both indicators can be predicted using runoff as follows:


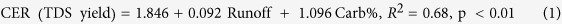



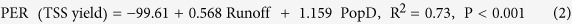


Concentrated rainfall in the monsoonal season causes tens or hundreds of times higher suspended solid concentration (SSC) in the high-flow period (Fig. 2a)^35^,^36^. This consequently enhances dissolution of detrital calcite that is concomitant with intensifying mechanical erosion of soils. Higher runoff therefore results in higher physical erosion and a higher rate of chemical weathering.

Significant coupling between TDS and TSS yields (fluxes) are found using the following linear/power law (R^2^ > 0.6, p < 0.01; Fig. 7n,o): TDS = aTSS + b or TDS = aTSS^b^. A similar observation was also reported for other rivers over Himalaya including the Mekong (TDS = 8.38TSS^31^; R^2 ^= 0.91, p < 0.01 at Pakse; TDS = 4.52TSS^0.28^; R^2 ^= 0.92, p < 0.01 at Chiang Saen; Fig. S6d) and Brahmaputra (CER = 4.61PER^0.44^)^35^. We conclude that runoff exerts a first-order control on chemical erosion, while the dissolution of detrital carbonate debris because of water erosion is another principal driver for chemical erosion. This process is particularly obvious in the Himalayan Rivers, enhanced runoff and higher relief boost their chemical and physical erosion rates (i.e., high specific fluxes of TDS and TSS).

In conclusion, huge data sets on the Mekong and other large rivers of the world are explored to gain new insights into how landuse, hydrology and lithology affect riverine solutes with special regard to HCO_3_^−^. Key drivers on historical changes of dissolved and particulate constituents show that clear spatial and inter-annual patterns are predominantly driven by hydrology, while human activities have altered the relative abundance of major ions. Decreases in Ca^2+^ and HCO_3_^−^ composition are due to irrigational precipitation of carbonates and increasing aquatic phytosynthsesis, while domestic discharge of salts and extensive agriculture in the basin have doubled the relative abundance of SO_4_^2−^, Cl^−^ and Na^+^. Conversely, significant declines in solute flux follow significant decreases in river discharge associated with water extraction for human activity.

Global analyses demonstrate that riverine HCO_3_^−^ concentration is correlated with river flow. The concentrations of HCO_3_^−^ (and other solutes) are also positively related to human land use, and inversely related to the area of forested land. However, fluxes of dissolved and particulate constituents are readily predicted using river discharge. Runoff, land cover and lithological composition can either positively, or negatively, contribute to dissolved and particulate yields. HCO_3_^−^ and TDS yields are predictable by runoff and percent of carbonate, while TSS yield is predicted by runoff and population density. Higher runoff results in higher physical erosion and, consequently, more intense chemical weathering. Therefore, enhanced runoff and higher relief prompt the chemical and physical erosion rates in the rivers over Himalaya. We thus conclude that the Himalayan Rivers are a disproportionally high contributor to global riverine carbon and other solute budgets. Our estimated global riverine HCO_3_^−^ flux is 34014 × 10^9 ^mol/y (0.41 Pg C/y), 3915 Mt/y for solute load including HCO_3_^−^, and 13553 Mt/y for TSS. Our study highlights the importance of riverine solute delivery in global carbon cycling.

## Methodology

### Study area

The 4800-km Mekong (8°52′′–22°53′N, latitude; 98°91′−108°99′E, longitude) rises at an elevation of 5200 m in the Qinghai-Tibet Plateau (QHTP), and then flows southward through Myanmar, Thailand, Laos, Cambodia and Vietnam. The Mekong River basin is generally divided into two sub-basins: the Upper Mekong Basin (UMB) covering an area of 195,000 km^2^ in China, and the Lower Mekong Basin (LMB) covering an area of 600,000 km^2^ downstream from China (Fig. S1).

The upper-most reaches of the Mekong are primarily fed by snowmelt in the Tibetan Highlands, while the broader catchment is characterised by a pronounced wet and dry season. The annual hydrograph of the Mekong is strongly seasonal as a result of south-western monsoonal driven seasonal precipitation (85%–90% of the annual rainfall arriving between May-October), and approximately 80% of the annual discharge occurs between June and November^11^,^39^. The river discharge usually begins rising in May and peaks in August or September, with the average peak flow at 25,195 m^3^/s (8140–43904 m^3^/s) over 1972- 1998 at Pakse. Around November, flows start receding and reach the lowest levels in February, at approximately 2156 m^3^/s (1554–3414 m^3^/s)^11^,^39^. The UMB (Lancang basin) in the Yuannan Province of China drains a warm semi-humid climate while the LMB has a tropical monsoonal climate with little variation of temperature ranging from 26 °C to 30 °C in the Delta.

The Mekong basin is dominated by forest and farm, respectively covering approximately 37% and 40% of the region. Shifting cultivation is practised both in the uplands and river valleys, while the Mekong Delta is a fertile rice-growing area throughout the year. The most common land cover type (agriculture) degrades the environment in a few major forms comprising irrigation, fertilizer application and use of pesticides, as well as deforestation for agricultural expansion. There are several cities (Vientiane and Phnom Penh) and small urban centres along the river networks, but most of the basin is classified as rural, with a low population density ranging from ~8 people/km^2^ in the mountainous region to ~570 people/km^2^ in the Delta^40^. The LMR supports a rapid population growth from a current 70 million to an expected 90 million by 2020. Accurate population data is not available, however, based on a growth rate of 1%, the population is estimated to have been 31.6 million in 1923, and 48.7 million in 1985. Alternatively, considering greater population expansion in the recent decades, the population can be estimated at 40.4 million in 1923 and 52.1 million in 1985 with an alternative growth rate of 0.5%. Agriculture will have to increase in order to meet the demands of a growing population, and irrigation and fertilizer use will become more intensive accordingly. The ongoing changes in population and economy within the basin will shift regional land use and consequently affect water quality. Aquaculture will also continue increasing as fish is the main source of protein for local people. Agricultural irrigation, the largest water consumption sector, consumes 42 km^3^ per year. Albeit industry is very scarce along the basin, while potential effects on water quality by industrial and municipal wastes from Phnom Penh and Vientiane are predicted. The solute deposition *via* precipitation is estimated at 2.9 × 10^9 ^mol/y for K^+^, 7.8 × 10^9^ mol/y for Na^+^, 6.9 × 10^9^ mol/y for Ca^2+^, 1.5 × 10^9^ mol/y for Mg^2+^, 9.8 × 10^9^ mol/y for Cl^−^, 2.5×10^9^ mol/y for SO_4_^2−^, 12.8×10^9^ mol/y for HCO_3_^−^, and 6.9 × 10^9^ mol/y for NO_3_^−11^.

Economic development and population growth are increasing the demand for electric power, and thus numerous projects of hydropower potential in the basin have been built or are planned in the near to medium future. For example, five dams (Manwan in 1992, Dachaoshan in 2003, Jinhong in 2009, Xiaowan in 2010 and Gongguoqiao in 2011) with a total capacity of 9300 MW are located along the upper Mekong main channel, which are part of the projected cascade of eight dams in China. Moreover, there are proposed 11 mainstream dams with 15,000 MW of power by Laos, and tens of large dams in the tributaries within Laos, Vietnam and Cambodia along the trans-boundary river are also planned^18^. These are altering traditional hydrological conditions and potentially solute including carbon, particularly TSS transports in the River. However, little information is available on historical delivery of solute from the Mekong to the oceans, even the well-documented TSS flux is mixed^12^,^13^,^14^. Our long-term riverine exports of dissolved and particulate matters will provide basic data to unravel the effects of human activities including dam construction on the riverine chemical and TSS loads.

The Mekong basin consists of a major Paleozoic-Mesozoic sedimentary terrain in the catchment in China and Lao PDR, and is mainly overlain by folded sedimentary and Precambrian metamorphics (sandstone, shale, schist, chert and limestone). Granite, basaltic exposures, and gabbros associated with an ancient structure zone (e.g., Paleozoic and Mesozoic sedimentary rocks) are also present in the upper basin. The river goes through a strip of Quaternary alluvium near Vientiane but then flows over Mesozoic sedimentary and metamorphic rocks, mainly composed of sandstone and mudstone, until it reaches northern Cambodia. From Cambodia downstream, an outcrop of Triassic sedimentary rocks and Neogene basalts is exposed in the alluvium. The detailed lithological map can be available in Gupta^41^, and lithological compositions are as follows: 8.4% sands and sandstones, 43.2% shales, 21.4% carbonate rocks, 18.2% shield rocks, 2.9% acid volcanic rocks and 5.8% basalts^4^.

### Data sources

The Mekong River Commission (MRC) composed of four member countries, Cambodia, Lao PDR, Thailand and Vietnam has established around 50 hydrochemical stations in the Mekong River basin to monitor concentrations of water quality parameters including nutrients, major dissolved ions, dissolved silica and other ancillary variables, as well as hydrological parameters. We are authorized to use the MRC water quality database (Metadata standard ISO 19115:2003/19139 from MRC dataset) for research purposes. In this study, TSS, alkalinity, major ions (Na^+^, K^+^, Ca^2+^, Mg^2+^, Cl^−^, SO_4_^2−^), dissolved silica (Si) measured at Chiang Saen and Pakse stations in the LMR over the period 1985–2011 were extracted. The sampling frequency was once per month, while bimonthly from 2009 onward. Alkalinity has been reported in the dataset, HCO_3_^−^ is considered equal to the alkalinity in the Mekong (pH > 7, mean: 7.7), based on calculations using Henry’s constant in the aquatic carbonate equilibrium. The daily water discharge over 1960–2007 at Chiang Saen, and 1923–2006 at Pakse was also available.

Water samples were collected at 50–100 cm below the surface, stored in refrigerators and analysed for chemical variables within 7 days of the time of collection. The collected water quality data were analysed following the acknowledged methods and submitted by various laboratories under authority of the MRC. All the laboratories have set up and implemented the quality assurance/quality control (QA/QC) system through inter-calibrations with the international standards and repeated analyses of samples for major elements. Recent works have confirmed the reliability of the data sets of major ions^11^,^39^.

Data for basin characteristics (land use/land cover, morphology, lithological composition, and hydrology) and solute species for the major, world rivers are compiled from international publications and open-access websites (for detailed information, see supplementary materials). Basin characteristics largely regulate HCO_3_^−^ and other solutes^4^,^30^,^33^, and therefore, were used to examine key drivers on concentrations, yields and fluxes of solute species for the world’s large rivers, which are poorly understood.

### Riverine fluxes of solute species and TSS

Solute and TSS exports are estimated at Chiang Saen, representing the dissolved and particulate loads from the upper Mekong River (Lancang basin), and Pakse station, the most downstream main-channel station with water discharge data (Fig. S1; Table S1). Thus, Pakse is used to quantify the riverine fluxes of solute and TSS from the Mekong River to the sea. The annual seaward fluxes are calculated using a most commonly-used extrapolation method based on regression models relating concentrations to discharge (Model 1) (Figs S2a and S3a). These models provide daily estimates of concentrations (C_i_), which are multiplied by the daily flow (Q_i_) to calculate the daily flux of solute including HCO_3_^−^ and TSS. Finally, annual flux is determined as follows:


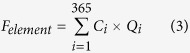


where *element* represents HCO_3_^−^, Na^+^, K^+^, Ca^2+^, Mg^2+^, Cl^−^, SO_4_^2−^, Si and TSS.

The rating relationship between element flux and instantaneous river discharge (Model 2) (Figs S2b and S3b) is used for the comparative quantifications of annual solute and TSS fluxes. Our selected two models are more reliable than the calculated fluxes using discharge-weighted concentration and the annual discharge in the Asian monsoonal rivers^42^. Our analyses indicate little difference in the riverine delivery of solute and TSS between the two models.


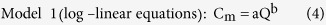



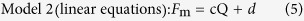


where C_m_ is the concentration of element m, Q is water discharge, a and d are the constants, and b and c are the regression coefficients. The models 1 and 2 are considered to be reliable through their comparisons with observed discharge-weight concentrations and fluxes (Tables S2 and S3). This has been corroborated by previous studies^43^. Nevertheless, due to the dilution effect for solute species during monsoonal freshet, slope values from linear relationship between daily solute flux and instantaneous river discharge are slightly higher without summer-autumn freshet sampling for major ions, which are prone to chemical-weathering control^11^. However, grab samples during peak flows are omitted (Table 1), this could result in the overestimation of riverine solute fluxes. For TSS, seasonal variations are opposite the solute species, thus, TSS exports could be under-estimated without peak-flow sampling.

Moreover, we provide the evidence of a good correlation between models and observed data to validate the modelling approach (flux at Chiang Saen station as an example; Fig. S4). Please note that instantaneous data rather than annual data are used for validation of models, because modelled annual averages are from daily data, while observed annual data are from monthly or bimonthly data. Thus, it is understandable that differences exist between the modelled data and observed data in Figs 2 and 3.

### Statistical analyses

Analysis of variance (ANOVA) was performed for differences of spatial concentrations and fluxes with significance at p < 0.05 by the least significant difference (LSD) test. Correlation analysis was employed to determine the relationships between concentration, specific flux (yield is flux divided by area) and flux of solute species (DIC and other major ions) and basin physical properties (lithology, hydrology, land use and anthropogenic activities, etc). Regression was further used to identify the basin physical and temporal controls on concentration, specific flux and flux, with concentration/areal yield/flux as dependent variables, while water discharge, year, lithology, land cover as independent variables. Statistical analyses were carried out at a significance level of p < 0.05 for each regressor^44^. The statistical processes were conducted using SPSS 15.0 and Sigmaplot 11.0.

## Additional Information

**How to cite this article**: Li, S. and Bush, R. T. Changing fluxes of carbon and other solutes from the Mekong River. *Sci. Rep.* **5**, 16005; doi: 10.1038/srep16005 (2015).

## Supplementary Material

Supplementary Information

## Figures and Tables

**Table 1:** Comparison of daily and instantaneous discharge (Q) ats Chiang Saen and Pakse stations (unit in m^3^/s).

	Size	Mean	Std. Dev	Std. Error	C.I. of Mean	Max	Min	Median	25%	75%
Chiang Saen										
Daily Q	17532	2700	2272	17	34	29300	338	1750	1020	3810
Instantaneous Q	141	2522	1939	171	338	11800	270	1770	1088	3565
Pakse										
Daily Q	30681	10137	10205	58	114	57800	1060	5097	2283	16133
Instantaneous Q	148	9131	8385	747	1478	32931	1410	5134	2267	15000
