# Supplementary material for: Changing fluxes of carbon and other solutes from the Mekong River
Source: Sci Rep. 2015 Nov 2;5:16005. doi: 10.1038/srep16005 (PMC4629185; doi:10.1038/srep16005)
Supplement: Supplementary Information [file srep16005-s1.pdf]

Changing carbon of the Mekong River

Siyue Li[1,2*], Richard T. Bush[1]

1. Southern Cross GeoScience, Southern Cross University, New South Wales, 2480, Australia

2. Key Laboratory of Reservoir Aquatic Environment, Chongqing Institute of Green and Intelligent Technology, Chinese Academy of Sciences, Chongqing 400714, China

Supplementary materials

Figs. S1-S9

Tables. S1-S5

References.

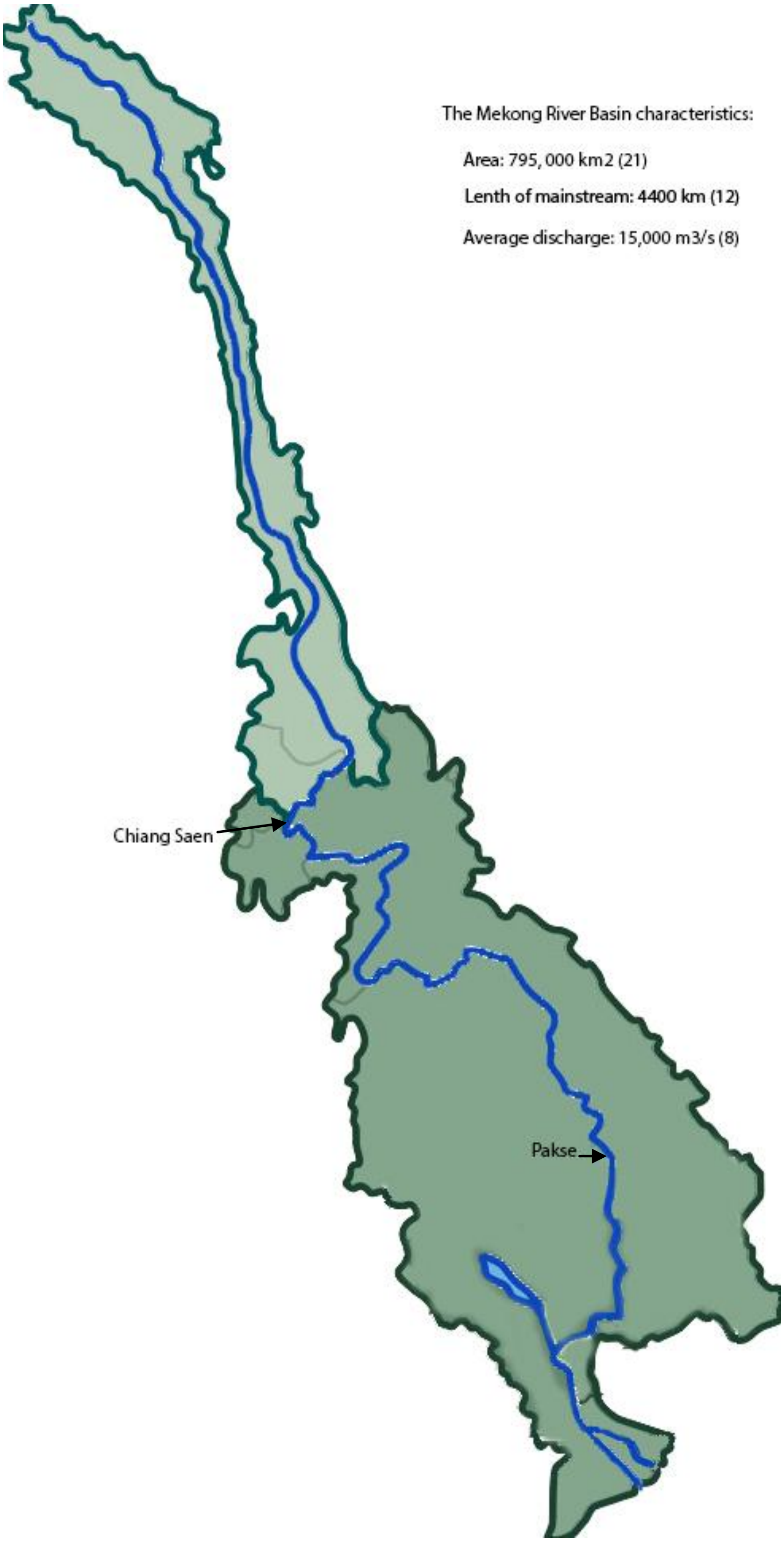

Fig. S1. Lower Mekong River showing main-channel stations (Chiang Saen represents the Upper Mekong catchment, while Pakse, the most downstream station with available water discharge, thus represents the whole basin) (This map is created using Photoshop CC 2014).

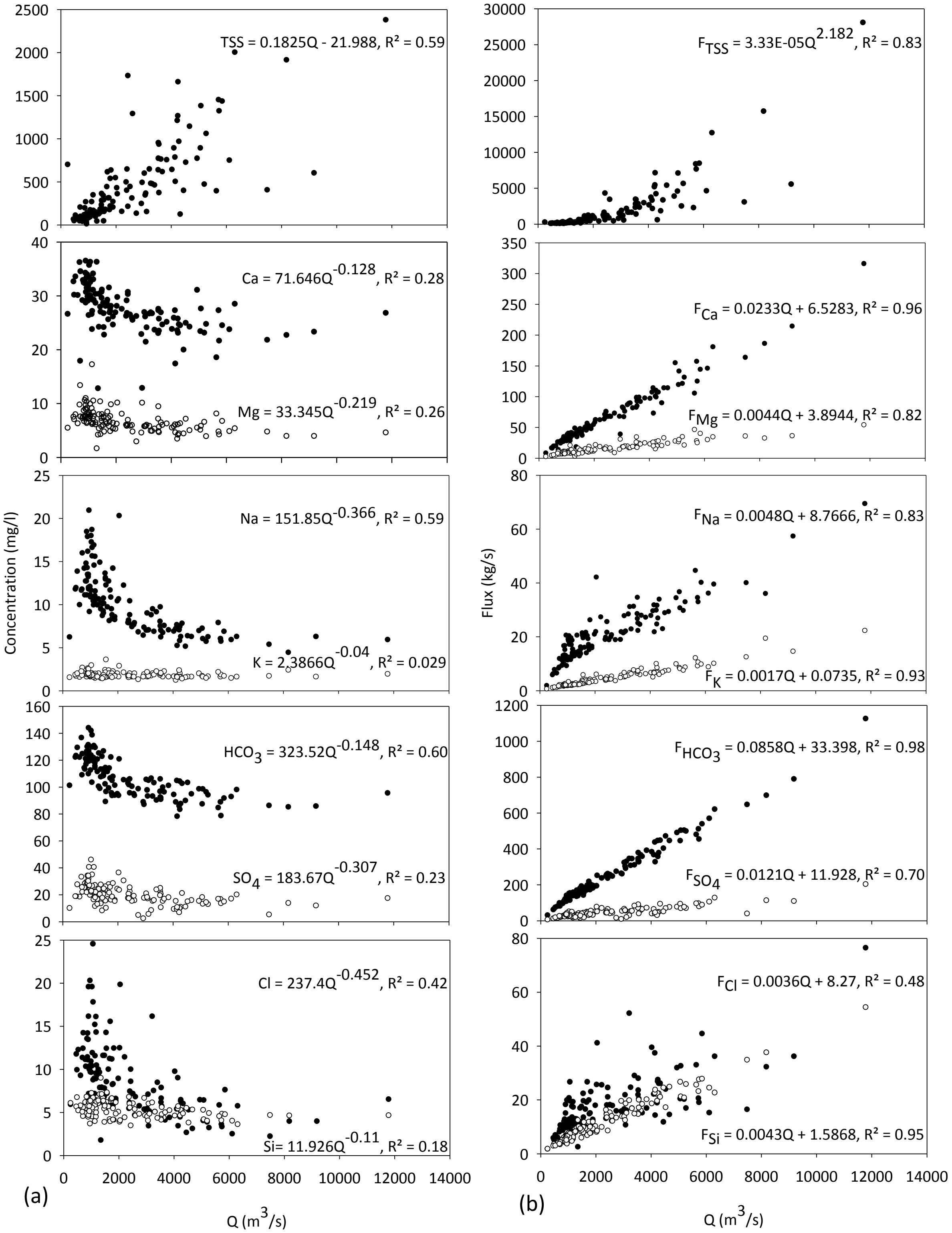

Fig. S2. Scatter plot s between instantaneous water discharge and concentrations (a) and fluxes (b) in the station Chiang Saen of Mekong River.

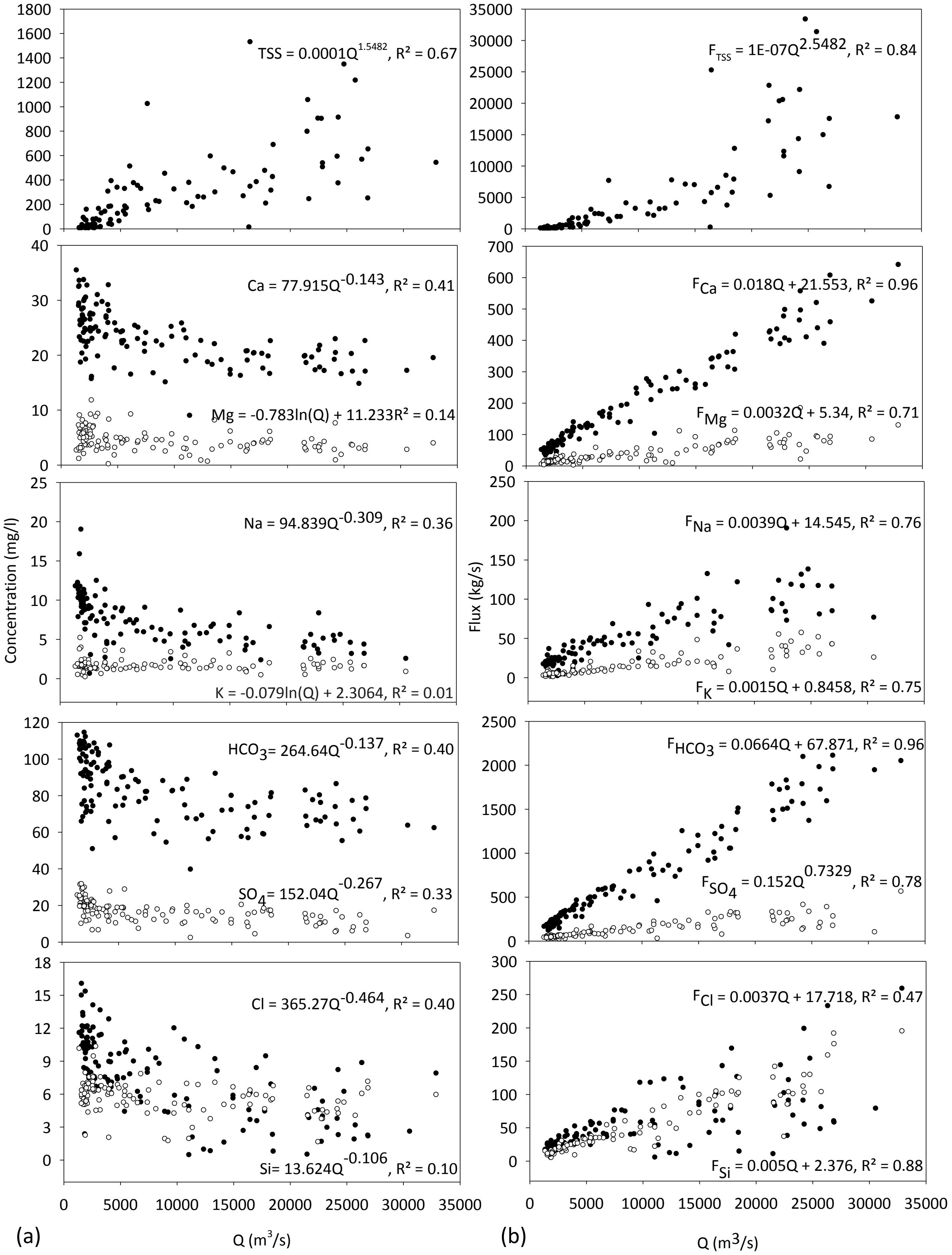

Fig. S3. Scatter plot s between instantaneous water discharge and concentrations (a) and fluxes (b) in the station Pakse of Mekong River.

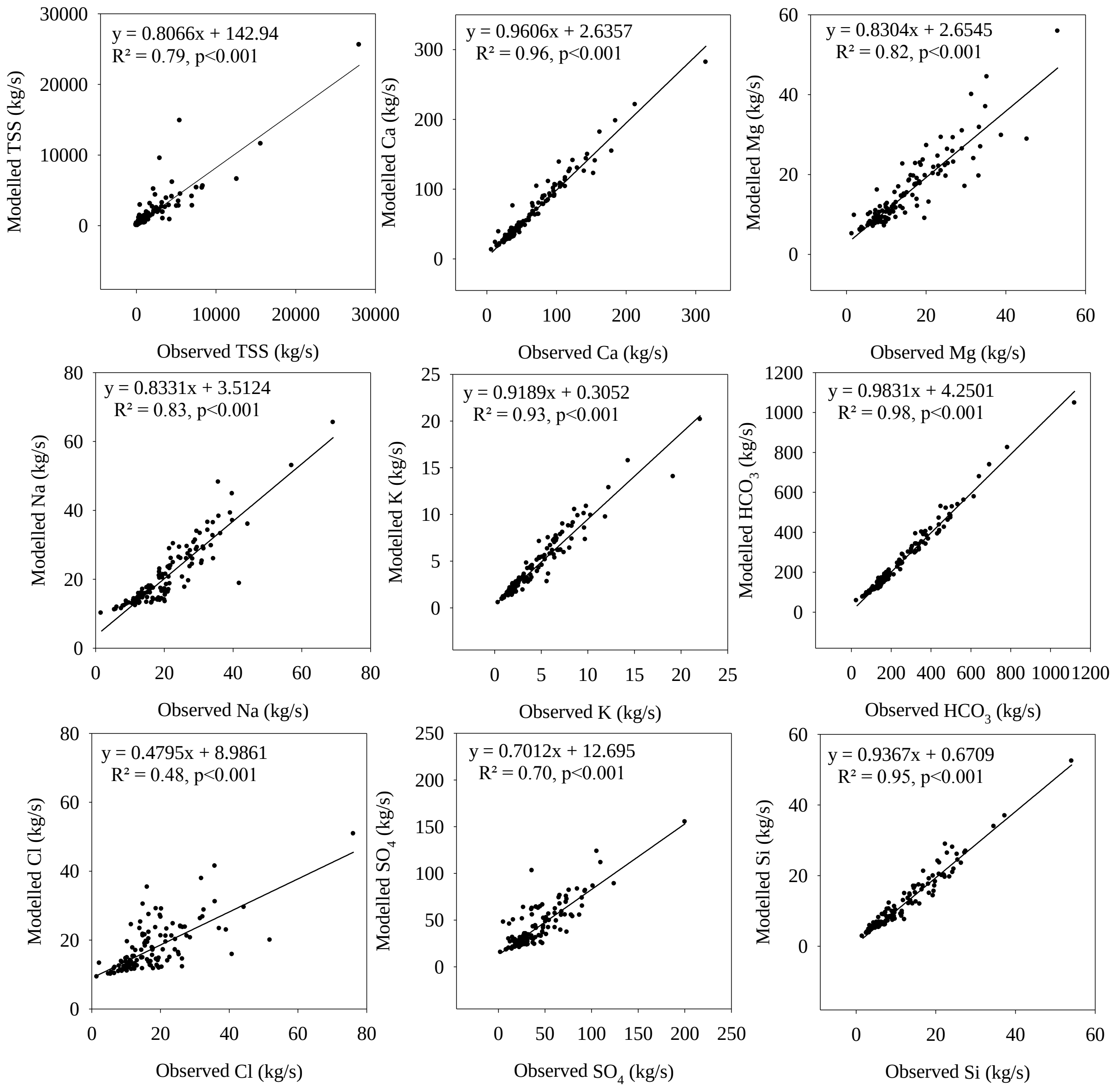

Fig. S4. Validation of modelling approach (comparison of instantaneous observed fluxes and modelled values, data are from station Chiang Saen).

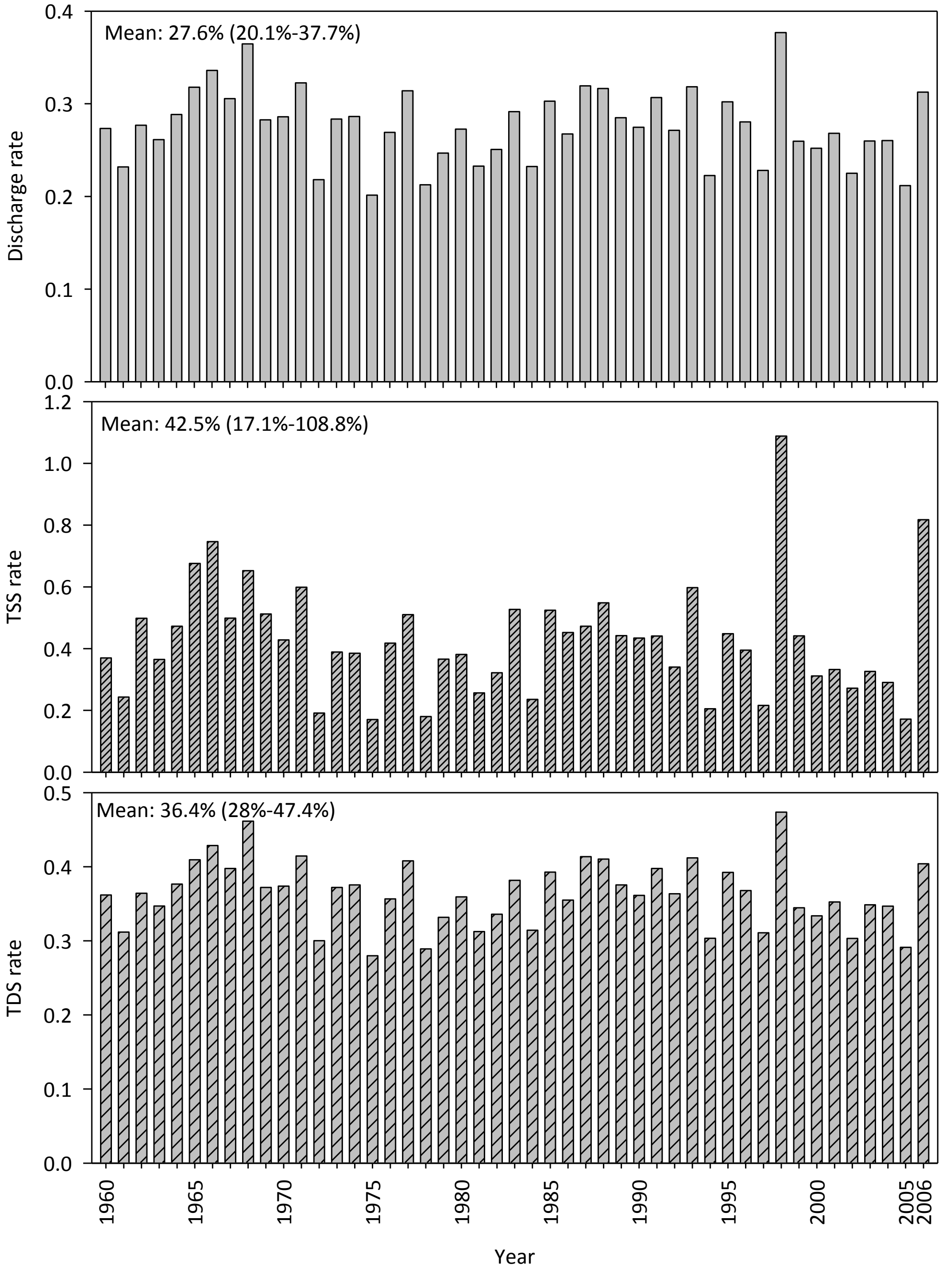

Fig. S5. The contributions of water discharge, TSS and TDS from upper catchment to the Mekong River (reflected by the ratio of the fluxes from Chiang Saen to Pakse).

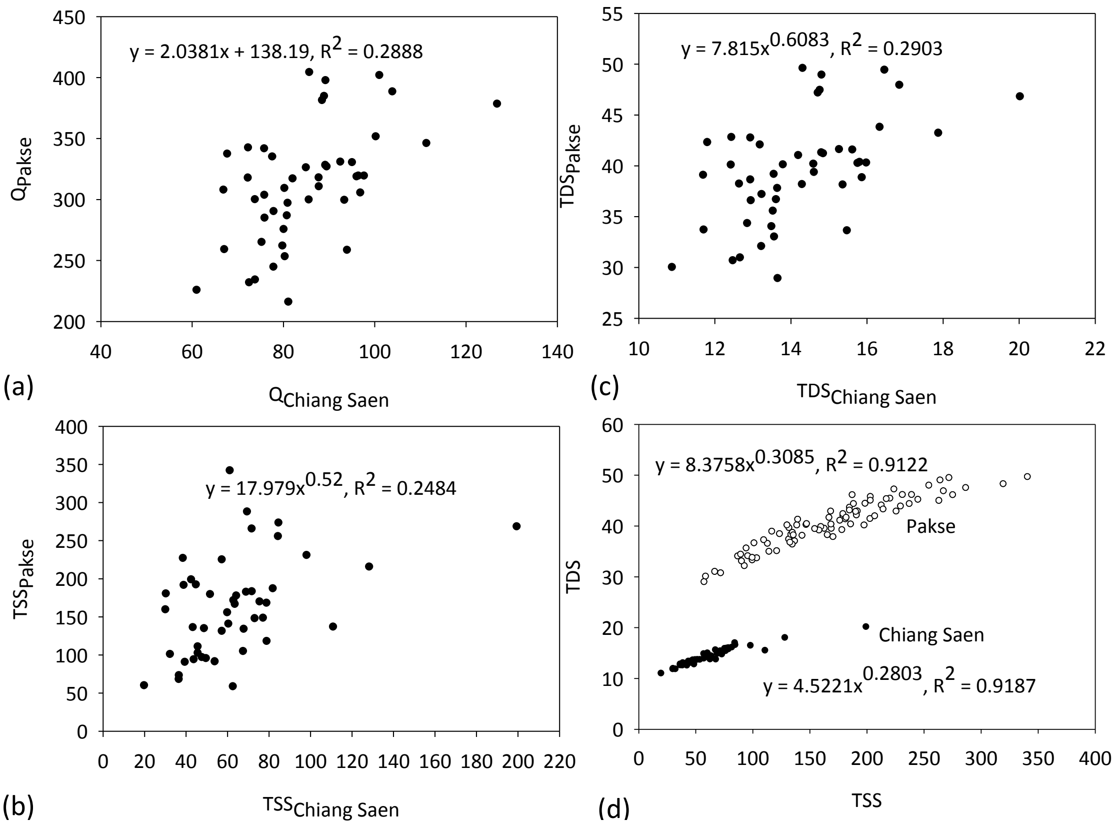

Fig. S6. The relationships between water discharge (a), TSS (b) and TDS (c) loads, as well as between TDS and TSS (d) at the both stations (Chiang Saen and Pakse) (unit in km^3/y for water discharge, Mt/y for TSS and TDS loads).

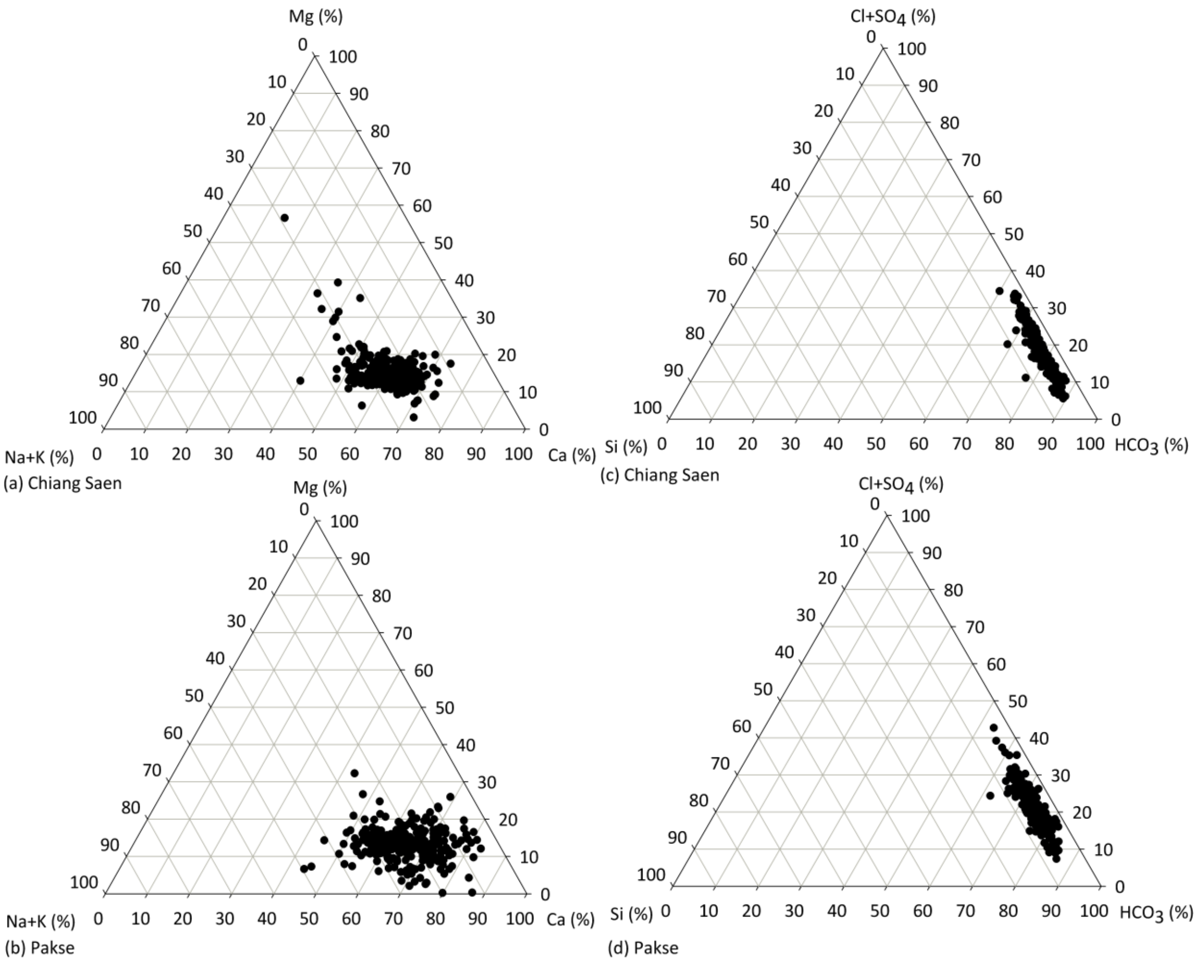

Fig. S7. Ternary plot showing relative abundance of cations (a and b) and anions (c and d) (unit in mg/L).

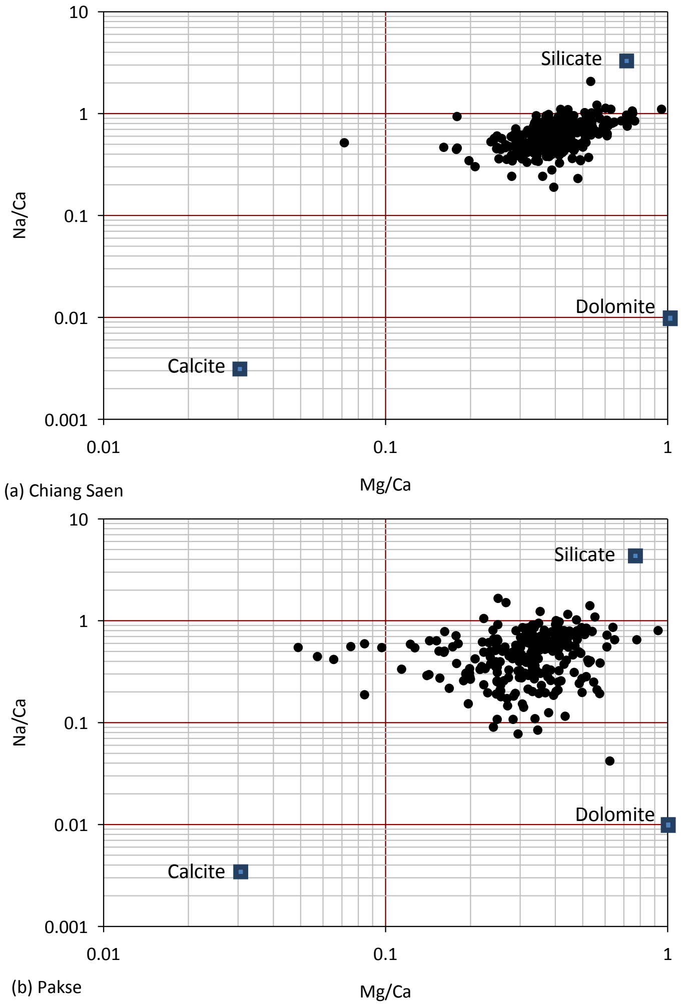

Fig. S8. Scatter plots between Na/Ca and Mg/Ca (molar ratio) in the stations (a-Chiang Saen, b-Pakse) of the LMR (End-members of rocks are from Gailardet et al., 1999 and Li et al., 2014).

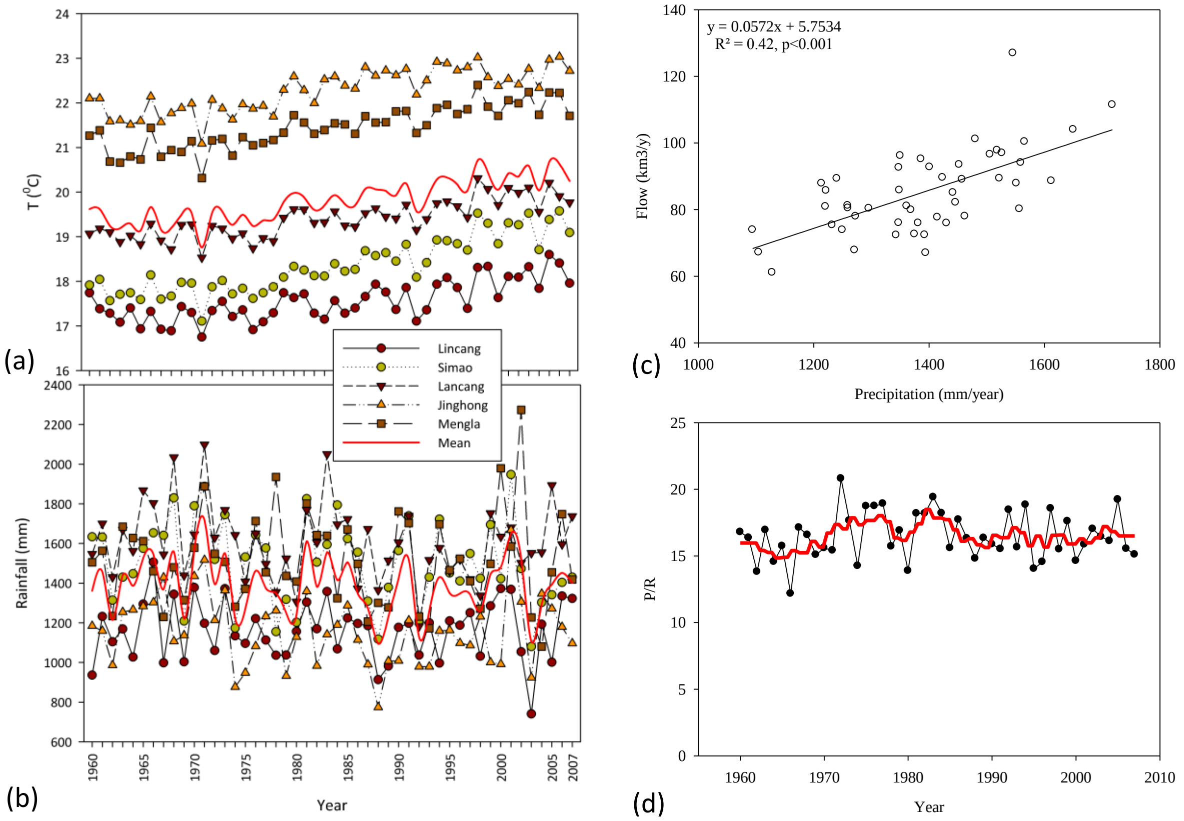

Fig. S9. Temperature (a) and rainfall (b) in five stations (Lincang, Simao, Lancang, Jinghong and Mengla) of upper Mekong River over 1960-2010. Mean temperature variations as year: T = 0.0284Year - 36.447, $R^2 = 0.72$, $p<0.01$. (c) Relationships between river discharge at Chiang Saen and annual mean precipitation in the upper basin, (d) Changes in the ratio of precipitation (P) to runoff (R) (P/R; unit in mm for precipitation, while km^3/year for runoff) in the upper basin (red line means smoothing analysis that averages the values at neighboring points).

Table S1. Main channel stations of the LMR included in this study

Stations		Country	Location		Distance from the River mouth	Drainage area	Pre-regulated period	Regulated period	Total
			latitude	longitude	km	$10^3 km^2$	1923-1991	1992-2007	
Chiang Saen[a]		Thailand	20.16.4	100.05.0	2363	189			
	Water discharge (km^3/y)						85.8	83.9	85.2
	TSS (Mt/y)						64.7	61.1	63.5
	TDS (Mt/y)						14.3	14.1	14.2
Pakse[b]		Lao PDR	17.07	105.48.0	869	545			
	Water discharge (km^3/y)						320.9	314.1	319.7
	TSS (Mt/y)						171.4	167.4	170.7
	TDS (Mt/y)						40.5	39.7	40.4

[a]data are from the time period of 1960-2007, [b]data are from the time period of 1923-2006.

Table S2. Comparison of annual mean concentrations in the LMR (unit in mg/L).

		TSS	Ca	Mg	Na	K	HCO_3	Cl	SO_4	Si	TDS
Chiang Saen											
Observed mean	Mean	317.53	27.76	7.14	9.96	1.86	108.08	9.41	20.58	5.56	188.51
	Max.	727.83	31.22	9.34	11.34	2.36	126.18	12.33	31.01	6.9	221.59
	Min.	63.24	23.98	4.7	8.24	1.48	93.62	7.42	8.99	4.7	167.45
	S.D.	174.94	1.87	1.16	0.8	0.26	7.26	1.32	5.32	0.51	11.85
Observed Q-weight mean	Mean	687.91	25.81	5.98	8.33	1.75	99.93	6.9	17.01	5	170.7
	Max.	1320.66	28.76	6.82	9.25	1.96	108.66	8.86	21.51	5.52	186.78
	Min.	293.8	23.29	4.68	7.69	1.6	93.45	5.65	13.52	4.32	161.16
	S.D.	350.03	1.77	0.63	0.53	0.13	4.84	1.03	2.52	0.33	8.39
Q-weight mean (predicted)	Mean	718.83	25.3	5.65	7.93	1.73	97.16	6.26	15.36	4.87	164.25
	Max.	1573	26.9	6.25	9.31	1.74	104.26	7.58	17.62	5.14	178.8
	Min.	329.51	23.55	5.01	6.54	1.72	89.49	4.97	13.04	4.58	148.9
	S.D.	208.99	0.6	0.22	0.5	0	2.64	0.48	0.83	0.1	5.38
Pakse											
Observed Mean	Mean	171.08	22.93	4.62	6.59	1.54	82.61	8.08	15.68	5.84	149.21
	Max.	509.6	28.06	5.91	9.28	3.05	94.91	12.3	29.4	7.46	164.14
	Min.	64.92	17.5	3.01	2.48	0.63	68.49	4.34	6.25	4.14	131.54
	S.D.	107.25	2.15	0.8	1.89	0.5	6.15	2	4.26	0.85	8.41
Observed Q-weight mean	Mean	478.45	20.56	3.78	5.13	1.45	74.44	5.47	12.84	5.28	129.24
	Max.	870.01	23.88	5.91	6.07	2.8	84.5	8.99	17.7	7	142.48
	Min.	175.15	19.08	1.98	2.14	0.51	63.9	3.56	5.49	3.49	119.39
	S.D.	205.51	1.33	1.01	1.18	0.56	5.73	1.51	3.58	1.05	6.82
Q-weight mean (predicted)	Mean	519.24	19.74	3.68	4.98	1.46	71.01	4.52	11.87	4.92	122.16
	Max.	844.84	21.01	4.02	5.69	1.47	75.37	5.5	13.32	5.15	131.53
	Min.	262.68	18.89	3.43	4.54	1.45	68.07	3.94	10.94	4.76	116.02
	S.D.	123.27	0.46	0.13	0.25	0.01	1.58	0.34	0.51	0.09	3.36

Observed mean is measured variables, Observed Q-weight mean is measured variables with water discharge weight, while Q-weight mean (predicted) is concentration derived from models.

Table S3. Statistics of dissolved and particulate material fluxes in the LMR.

	Discharge	Flux (Mt/y)																
	km^3/y	TSS_1	Ca_1	Ca_2	Mg_1	Mg_2	Na_1	Na_2	K_2	HCO_{31}	HCO_{32}	Cl_1	Cl_2	SO_{41}	SO_{42}	Si_1	Si_2	TDS_2
Chiang Saen																		
Mean	85.21	63.52	2.15	2.19	0.48	0.50	0.67	0.69	0.15	8.25	8.36	0.53	0.53	1.30	1.41	0.41	0.42	14.24
Max.	126.98	199.74	2.99	3.17	0.64	0.68	0.83	0.89	0.22	11.36	11.95	0.63	0.63	1.66	1.91	0.58	0.60	20.04
Min.	61.11	20.14	1.64	1.63	0.38	0.39	0.57	0.57	0.11	6.37	6.30	0.46	0.47	1.08	1.12	0.31	0.31	10.89
S.D.	12.40	29.25	0.26	0.29	0.05	0.05	0.05	0.06	0.02	0.97	1.06	0.04	0.04	0.12	0.15	0.05	0.05	1.73
Pakse																		
Mean	319.69	170.69	6.29	6.43	1.17	1.19	1.58	1.71	0.47	22.63	23.37	1.43	1.74	3.77	3.77	1.57	1.67	40.35
Max.	403.85	341.19	7.68	7.95	1.41	1.46	1.86	2.03	0.59	27.65	28.96	1.63	2.05	4.49	4.48	1.93	2.09	49.55
Min.	215.55	57.84	4.53	4.56	0.87	0.86	1.23	1.30	0.32	16.25	16.45	1.19	1.36	2.87	2.87	1.11	1.15	28.86
S.D.	43.76	60.31	0.73	0.79	0.12	0.14	0.15	0.17	0.06	2.65	2.91	0.10	0.16	0.37	0.37	0.19	0.22	4.82

[1]represents model1

[2]represents model 2

Table S4. Comparison of global watershed physical characteristics, concentrations, fluxes and yields of DIC and major solutes.

(a) Lithology (data are from Amiotte Suchet et al., 2003)

		Area 10^3 km^2	Discharge km^3/y	Runoff mm	Sands and sandstone %	Shales %	Carbonate %	Shield %	Acid Volcanic %	Basalts %
1(a)	Amazon (upper)	5830	6223	1067.4	16.7	51	3.9	27	1.9	0
1(b)	Amazon (lower)	5830	6223	1067.4	16.7	51	3.9	27	1.9	0
2	Amur	1870	407	217.6	11.9	28	0	54	0	6.5
3	Brahmaputra	583	612							
4	Chao Phraya	160	30.4	190						
5	Colorado	670	28	41.8	55.6	0	0	10	34	0
6	Columbia	620	269	433.9	1.4	13	0	44	33.4	8.4
7	Danube	740	140	189.2	3.3	67	14.5	16	0	0
8	Don	390	31	79.5	0	94	5.9	0	0	0
9	Fraser	240	104	433.3	0	69	0	22	9.5	0
10	Ganges	1060	459							
11(a)	Ganges-Brahmaputra	1643	1071	651.9	15.4	32	33.8	18	0	1.4
11(b)	Ganges-Brahmaputra	1643	1071							
12	Godavari	300	147	490	3.9	0	0	62	0	34.5
13	Yenisei	2440	665	272.5	6.4	12	6.9	38	0	36
14	Indigirka	340	54	158.8	39.9	60	0	0	0	0
15	Indus	880	251	285.2	16.8	24	26	33	0	0
16	Irrawaddy	400	459	1147.5	30.6	14	44	11	0	0
17	Kolyma	590	122	206.8	72.3	28	0	0	0	0
18	Lena	2320	393	169.4	10.8	39	11.2	34	0	5.1
19	Limpopo	310	27	87.1	25	0	14.2	39	0	21.6
20	Mackenzie	1470	260	176.9	0	54	20.6	26	0	0
21	Magdalena	260	313	1203.8	23.8	29	4.8	24	19.1	0
22(a)	Mekong (Chiang Saen)	189	85.2	450.8						

22(b)	Mekong (Pakse)	545	319.7	586.6	8.4	43	21.4	18	2.9	5.8
22(c)	Mekong	820	623	759.8	8.4	43	21.4	18	2.9	5.8
23	Mississippi	3130	570	182.1	25.3	48	18.1	8.7	0.3	0
24	Murray Darling	1110	40	36	0.9	72	0	21	2.7	2.9
25	Niger	1500	166	110.7	57.8	0	6.3	35	0	0.8
26	Nile	1840	125	67.9	31.9	0	2.5	45	0	20.4
27	Ob	3010	477	158.5	19.8	72	2.7	2.7	0	3
28	Orange	660	26	39.4	68.8	0	9.8	17	0	4.8
29	Orinoco	960	759	790.6	17.7	47	1.3	30	0	3.8
30	Parana	2840	666	234.5	27.3	44	1.2	15	0.8	12.3
31	Red	120	123	1025						
32	Salween	325	211	649.2						
33	Sao Francisco	590	119	201.7	14	8	39.8	38	0	0
34	Senegal	360	15	41.7	64.5	3.2	0	29	0	3.3
35	Severnaia Dvina	300	97	323.3	10.9	78	10.8	0	0	0
36	Xijiang	352	230	653.4	0	0	82.4	18	0	0
37	St. Lawrence	870	360	413.8	0	6.1	24.9	69	0	0
38	Tigris-Euphrates	930	156	167.7	17.2	26	42.5	2.2	11.3	1
39	Yana	210	25	119	50.6	49	0	0	0	0
40	Yangtze	1740	908	521.8	13.9	7.9	44	34	0	0
41	Yellow	750	33	44	27.6	5.9	7.6	59	0	0
42	Yukon	780	169	216.7	0	85	0	15	0	0
43	Zaire	3600	1298	360.6	46.6	0	10.1	42	0	1.4
44	Zambesi	1310	109	83.2	45.9	0	13.6	39	0	1.8
World total		148170	37400	252.4	26.3	25.3	13.4	27.6	2.3	5.1

(b) Land cover composition (data are mainly from Ludwig et al., 1996 and http://multimedia.wri.org/watersheds_2003/)

	FTSS	Elev.	Slope	SoilH	APPT	AT	Veg C	SoilC	NPP	PopD	FDOC	FPOC	POC%	DOC	POC	Land cover (%)						
	t/km2/yr	m	radian	cm	mm	0C	kg/m2	kg/m3	kg/m2	h/km2	t/km2/yr	t/km2/yr	% tss			Forest	Grass, Savanna And Shrub	Vegetation	Wetland	Cropland	Irrigated Cropland	Urban
														mg/L	mg/L							
1(a)																						
1(b)	190	455	0.0434	226	2084	24.4	11.7	13.3	0.669	4	4.461	2.826	6.09	4.46	2.83	73.4	10.2	83.6	8.3	14.1	0.1	0.6
2	28	571	0.0699	137	479	-1.6	6.9	12.7	0.472	33						53.8	8.8	62.6	4.4	18.4	0.8	2.6
3										182						18.5	44.7	63.2	20.7	29.4	3.7	2.4
4										119						35.4	11.1	46.5	8.4	44.7	12.5	9.2
5	190	1505	0.1718	176	283	11.7	4.2	6.6	0.322	9						17	74.9	91.9	2.5	0.9	2	6.9
6	22	1336	0.2197	232	482	7	8.5	10.7	0.442	16	0.795	0.098		2.12	0.26	50	35.5	85.5	6.3	6.4	3.6	7.3
7	83	514	0.125	154	766	9.1	4.1	10.7	0.577	125						18.2	3.2	21.4	1.4	66.9	5.2	10.7
8	18	138	0.0147	234	434	6.2	1.2	8.4	0.5	47	0.6	0.252		8.81	3.69	1.4	5.4	6.8	0.5	83.1	3.2	8.8
9	91	1173	0.2555	178	606	4.5	14.2	12	0.585	4						85.7	6.2	91.9	1.7	0.4	0	4.1
10										401						4.2	13.4	17.6	17.7	72.4	22.7	6.3
11(a)	668	1406	0.1353	153	1443	20.3	3.7	11	0.477	316	2.215	5.222		3.87	9.13	9.8	25.6	35.4	18.9	55.6	15.3	4.8
11(b)																						
12	550	399	0.0433	154	1132	26.3	3.8	8.6	0.568	260						6.8	22.5	29.3	1.2	64	11.7	6.7
13	5	769	0.0855	93	356	-4.5	7.6	12.6	0.377	3						39.7	32.4	72.1	2.7	12.8	0	1.3
14	39	738	0.1261	37	230	-14.5	3.6	13.1	0.241	1						2.4	69.7	72.1	3	0	0	0.1
15	260	1671	0.1476	92	506	18.5	2.2	9.3	0.331	165	2.929	1.794	0.46	14.4	8.82	0.4	46.4	46.8	4.2	30	24.1	4.6
16	620	745	0.1477	242	1825	22.4	8.5	9.7	0.631	79						56.2	9.7	65.9	6.3	30.5	3.4	1.9
17	9	590	0.1195	24	267	-10.9	3.7	12.8	0.246	1						0.7	45.3	46	1	0	0	0.3
18	5	608	0.0857	49	337	-8.6	6	12.9	0.318	1						64.7	11.4	76.1	0.6	1.7	0	0.4
19	80	795	0.0498	189	645	21	3.2	6.8	0.434	32						0.7	67.7	68.4	2.8	26.3	0.9	4.5
20	23	634	0.0964	184	339	-4.2	8.2	12.4	0.375	1	0.838	0.855	2.95	4.93	5.03	66	14.7	80.7	48.9	2.6	0	1.9
21	920	1224	0.2527	194	1934	23.7	5.3	12.2	0.548	83						37.2	14.8	52	0.2	35.8	2.4	10.3

22(a)																					
22(b)	200	854	0.0858	210	1644	22.5	8.1	10.5	0.609	71	2.81	1.18	2.2	4.75	2	41.5	17.2	58.7	8.7	37.8	2.9	2.1
22(c)	200	854	0.0858	210	1644	22.5	8.1	10.5	0.609	71	2.81	1.18	2.2	4.75	2	41.5	17.2	58.7	8.7	37.8	2.9	2.1
23	120	646	0.0497	214	776	10.3	3.1	11.1	0.487	27	1.319	0.32		8.79	2.14	22.2	28.5	50.7	20	35.8	3.1	12.7
24	29	272	0.0337	161	469	18.5	4	7.8	0.444	6						8	62.1	70.1	3.4	28.4	1.6	1.2
25	33	337	0.0303	294	1068	27.4	3.1	8.8	0.467	43	0.593	0.414	3.27	3.71	2.59	0.9	68.6	69.5	4.1	4.4	0.1	0.5
26	40	892	0.0577	221	944	24.3	2.8	8	0.454	46	0.089	0.116		2.95	3.85	2	53	55	6.1	10.7	1.4	1
27	6	305	0.0345	216	393	-0.6	4.4	20	0.406	10	1.182	0.115		9.09	0.88	33.9	16	49.9	11.2	36.9	0.5	3
28	100	1255	0.0484	160	362	18.1	1.4	4.8	0.318	10	0.25	0.106	2.18	2.5	1.06	0.2	85	85.2	0.8	6	0.5	2.2
29	150	347	0.0549	228	2359	26.1	8.2	11.2	0.592	17						50.5	37.8	88.3	15.3	7.6	0.2	2.6
30	30	503	0.0443	228	1155	21.5	3.3	15	0.424	26	1.432	0.279	2.64	8.68	1.69	18.1	33	51.1	10.9	43.3	0.5	4.2
31	1100	897	0.1315	306	1597	21	7.7	9.2	0.605	191						43.2	15.5	58.7	5.4	36.3	3.9	2.1
32																43.4	48.3	91.7	9.5	5.5	0.4	0.5
33	9	627	0.0449	261	1141	23.1	4	6.1	0.468	45						3.1	31.8	34.9	9.7	60.2	0.3	2.8
34	8	223	0.0216	133	706	28.4	2.7	5.7	0.466	10		0.072			1.5	0.1	68.2	68.3	3.6	4.8	0	0.1
35	13	127	0.0141	342	474	0	8.1	14.4	0.466	6	4.498	0.197		13.63	0.6	83.4	0.4	83.8	1.5	11.7	0	3
36	149	598	0.0805	198	1501	19.8	2.8	6.8	0.463	194						9.6	6.1	15.7	1.3	66.5	5.2	5.3
37	4	274	0.0407	210	837	4.4	5.8	10.9	0.519	59	1.632	0.326	5.98	3.75	0.75	43.5	0.1	43.6	47.2	16.4	0.2	14.5
38	57	625	0.0933	200	282	18.6	1.6	5.5	0.332	57						1.2	47.7	48.9	2.9	25.4	9.1	6.2
39	14	746	0.129	30	129	-14.8	3.5	13.5	0.235													
40	250	1527	0.1321	196	1074	14.4	5.3	10.4	0.457	226	5.69	6.144	2.52	12.37	13.36	6.3	28.2	34.5	3	47.6	7.1	3
41	1400	1838	0.1197	179	485	9.2	2	8.5	0.367	156	0.481	14.678	0.7	6.25	190.63	1.5	60	61.5	1.1	29.5	7.2	5.9
42	71	748	0.1468	219	337	-4.9	4.2	14.7	0.251	1	0.953	0.307	0.32	4.14	1.33	64	27.6	91.6	27.8	0	0	0.4
43	11	775	0.037	310	1520	24	8.4	12.5	0.603	15	2.465	0.68	7	7.25	2	44	45.4	89.4	9	7.2	0	0.2
44										18						4	72	76	7.6	19.9	0.1	0.7

(c) Concentrations (data without reference are from Gaillardet et al., 1999)

	pH	TDS mg/L	TSS mg/L	Na µmol/L	K µmol/L	Ca µmol/L	Mg µmol/L	Cl µmol/L	SO_4 µmol/L	HCO_3 µmol/L	SiO_2 µmol/L	T_z^+	Source
1(a)		122		278	28.9	477	96.7	183	73	1122	185	1454.3	Stallard and Edmond, 1983
1(b)		38	182	63.4	20.8	129	39.8	30.5	17.8	323	120	421.8	Stallard and Edmond, 1983
2		55	72	126	26	223	95	66	65	477	36	788	
3	7.6	110	1060	100	50	390	170	20	60	1110	160	1270	Galy and France-Lanord, 1999
4													
5													
6		115	64	217	28	450	206	86	115	1033	150	1557	
7		428	313	739	51	1473	1117	1509	660	3328	69	5970	
8		829	80	6348	26	2073	1481	4200	2240	3295	5	13482	
9													
10	7.6	130	1100	180	70	470	200	90	80	1420	130	1590	Galy and France-Lanord, 1999
11(a)		196		613	69	710	496	169	146	2684	158	3094	Sarin et al., 1989
11(b)													Galy and France-Lanord, 1999
12		193	1619	352	56	755	99	403	104	1721	352	2116	
13		112	23	226	26	413	152	271	104	939	138	1382	
14		34				137.5	66.667	70.423	96.875	165.574		408.334	SCOPE 42 - Biogeochemistry of Major World Rivers
15	8.21	158		191	68	637	210	73	313	1060	71	1953	Karim and Veizer, 2000
16		201	535	1304	51	250	247	514	52	1967	167	2349	
17		52				290	275	8.451	50	472.131		1130	SCOPE 42 - Biogeochemistry of Major World Rivers
18		92		74	10	360	141.5	25	72	867	67	1087	Huh et al., 1998a
19		238	1269	896	118	483	506	406	54	2361	295	2992	
20	7.5	209	134	330	26	890	337	226	369	1803	50	2810	

21		118	928	361	49	375	136	383	150	808	210	1432	
22(a)	7.6	164.3	718.8	344.7	44.3	632.4	235.3	176.5	160.0	1592.7	174.0	2124.44	This study
22(b)	7.7	122.2	519.2	216.6	37.4	493.5	153.1	127.2	123.6	1164	175.6	1547.2	This study
22(c)		263	321	663	48	1001	367	448	343	2305	167	3447	
23		216	862	478	72	850	366	294	266	1902	127	2982	
24		453	1271	4391	154	525	700	4886	396	1541	83	6995	
25		59	260	78	28	138	78	26	5	549	233	538	
26		388	1400	2261	200	775	576	1257	542	2852	213	5163	
27		126	40	230	26	465	210	186	89	1279	43	1606	
28													
29		27	132	61	21	70	20.5	251	30	110	50	263	Nemeth et al., 1982
30		86	139	231	94	173	86	185	21	690	285	843	
31	8.2	172	1057	54	24	842	151	17	32	1968	169	2064	Moon et al., 2007
32		306		435	26	1150	658	571	10	3475		4077	
33													
34													
35													
36	7.76	185		259	47	791	271	69	125	2033	115	2430	Zhang et al., 2007
37		168	12	239	35	750	247	214	146	1656	40	2268	
38	8.33	277		279.6	36.9	1165.3	380.8	583.1	241.7	2521.3	211.7	3408.7	Varol et al., 2013
39		30				160	137.5		7.292	273.77		595	SCOPE 42 - Biogeochemistry of Major World Rivers
40	7.8	202	520	325	59	807.5	346	161	177	2110	103	2691	Chen et al., 2002
41	8	491	26900	2207	401	1115	1090.5	1797	999	3208		7019	Chen et al., 2005
42		183	286	113	31	795	295	31	233	1787	128	2324	
43		33	32	87	33	110	70	76	41.5	116	161	480	Meybeck, 1984
44		80	194	235	49	265	169	186	31	525	280	1152	
WSM		127		148	26	500	187.5	96	109.5	1256	134	1549	Meybeck, 2004
WDWA		97		240	44	297	122.5	167	87.5	798	145	1123	Meybeck, 2004

WSM-World spatial median; WDWA-World discharge-weighted average.

(d) Fluxes

	TDS Mt/y	TSS Mt/y	Na 10^9mol/y	K	Ca	Mg	Cl	SO_4	HCO_3	SiO_2	Source
1(a)											Stallard and Edmond, 1983
1(b)	236.5	1107.7	394.5	129.4	802.8	247.7	189.8	110.8	2010	746.8	Stallard and Edmond, 1983
2	22.4	52.4	51.3	10.6	90.8	38.7	26.9	26.5	194.1	14.7	
3	56	648.7	55	29	211	94	19	91	473	78	Galy and France-Lanord, 1999
4		10.9									
5		127.3							44.9		
6	30.9	13.6	58.4	7.5	121.1	55.4	23.1	30.9	277.9	40.4	
7	59.9	61.4	103.5	7.1	206.2	156.4	211.3	92.4	465.9	9.7	
8	25.7	7	196.8	0.81	64.3	45.9	130.2	69.4	102.1	0.16	
9		21.8							93.6		
10	68.6	504.9	128	29	252	93	40	39	713	59	Galy and France-Lanord, 1999
11(a)	209.9	1097.5	656.5	73.9	760.4	531.2	181	156.35	2874.6	169.2	Sarin et al., 1989
11(b)	124.6		183	59	462	187	59	130	1185	137	Galy and France-Lanord, 1999
12	28.4	165	51.7	8.2	111	14.6	59.2	15.3	253	51.7	
13	74.5	12.2	150.3	17.3	274.6	101.1	180.2	69.2	624.4	91.8	
14	1.8	13.3			7.4	3.6	3.8	5.2	8.9		SCOPE 42 - Biogeochemistry of Major World Rivers
15	39.7	228.8	47.9	17.1	159.9	52.7	18.3	78.55	266.1	17.8	Karim and Veizer, 2000
16	92.3	248	598.5	23.4	114.8	113.4	235.9	23.9	902.9	76.7	
17	6.3	5.3			35.4	33.6	1	6.1	57.6		SCOPE 42 - Biogeochemistry of Major World Rivers
18	36.2	11.6	29.1	3.9	141.5	55.6	9.8	28.3	340.7	26.3	Huh et al., 1998
19	6.4	24.8	24.2	3.2	13	13.7	11	1.5	63.7	8	
20	54.3	34.8	85.8	6.8	231.4	87.6	58.8	95.9	468.8	13	

21	36.9	239.2	113	15.3	117.4	42.6	119.9	47	252.9	65.7	
22(a)	14.2	63.5	29.1	3.8	53.7	19.9	14.9	13.5	135.2	14.8	This study
22(b)	40.4	170.7	68.8	11.9	157.3	48.7	40.3	39.3	371	56	This study
22(c)	163.8	164	413	29.9	623.6	228.6	279.1	213.7	1436	104	
23	123.1	375.6	272.5	41	484.5	208.6	167.6	151.6	1084.1	72.4	
24	18.1	32.2	175.6	6.2	21	28	195.4	15.8	61.6	3.3	
25	9.8	49.5	12.9	4.6	22.9	12.9	4.3	0.8	91.1	38.7	
26	48.5	73.6	282.6	25	96.9	72	157.1	67.8	356.5	26.6	
27	60.1	19.1	109.7	12.4	221.8	100.2	88.7	42.5	610.1	20.5	
28		66							17.8		
29	20.5	144	46.3	15.9	53.1	15.6	190.5	22.75	83.5	38	Nemeth et al., 1982
30	57.3	92.6	153.8	62.6	115.2	57.3	123.2	14	459.5	189.8	
31	21.2	130	6.6	3	103.6	18.6	2.1	3.95	242.1	20.8	Moon et al., 2007
32	64.6		91.8	5.5	242.7	138.8	120.5	2.1	733.2		
33		5.3							188.8		
34		2.9							2.5		
35		3.9							239.4		
36	42.6	52.4	59.6	10.8	181.9	62.3	15.9	28.75	467.6	26.5	Zhang et al., 2007
37	60.5	4.3	86	12.6	270	88.9	77	52.6	596.2	14.4	
38	43.1	53	43.6	5.8	181.8	59.4	91	37.7	393.3	33	Varol et al., 2013
39	0.75	2.9			4	3.4		0.18	6.8		SCOPE 42 - Biogeochemistry of Major World Rivers
40	183.4	472.2	295.1	53.6	733.2	314.2	146.2	160.7	1915.9	93.5	Chen et al., 2002
41	16.2	887.7	72.8	13.2	36.8	36	59.3	32.95	105.9		Chen et al., 2005
42	30.9	48.3	19.1	5.2	134.4	49.9	5.2	39.4	302	21.6	
43	42.8	41.5	112.9	42.8	142.8	90.9	98.6	53.85	150.6	209	Meybeck, 1984
44	8.7	45.9	25.6	5.3	28.9	18.4	20.3	3.4	57.2	30.5	
Sum	2103.9	7409.8	4862.8	778.9	7409.8	3391.9	3181.4	1887.4	20209.8	2500.6	
World total	3618.2	12610	8460	1240	12500	5230	6120	2060	31900	6480	Glay and France-Lanord, 1999

Fluxes are calculated by multiplying concentrations by annual discharge.

(e) Specific flux

		TDS	TSS	Na	K	Ca	Mg	Cl	SO_4	HCO_3	SiO_2
		$t/km^2/y$	$t/km^2/y$	10^3 $mol/km^2/y$							
1(a)	Amazon (upper)										
1(b)	Amazon (lower)	40.6	190	67.7	22	138	42.5	32.6	19	344.8	128
2	Amur	12	28	27.4	5.7	48.5	20.7	14.4	14.1	103.8	7.8
3	Brahmaputra	96.1	1112.7	94.3	50	362	161	32.6	156.1	811.3	134
4	Chao Phraya		68								
5	Colorado		190							67	
6	Columbia	49.9	22	94.2	12	195	89.4	37.3	49.9	448.2	65
7	Danube	81	83	139.8	9.6	279	211	286	124.9	629.6	13
8	Don	65.9	18	504.6	2.1	165	118	334	178.1	261.9	0.4
9	Fraser		91							390	
10	Ganges	64.7	476.3	120.8	27	238	87.7	37.7	36.8	672.6	55.7
11(a)	Ganges-Brahmaputra	127.8	668	399.6	45	463	323	110	95.2	1750	103
11(b)	Ganges-Brahmaputra	75.8		111.4	35.9	281.2	113.8	35.9	79.1	721.2	83.4
12	Godavari	94.6	550	172.5	27	370	48.5	198	51.0	843.3	172
13	Yenisei	30.5	5	61.6	7.1	113	41.4	73.9	28.3	255.9	38
14	Indigirka	5.4	39			21.8	10.6	11.2	15.4	26.3	
15	Indus	45.1	260	54.5	19	182	59.9	20.8	89.3	302.3	20
16	Irrawaddy	230.6	620	1496.3	59	287	283	590	59.7	2257	192
17	Kolyma	10.8	9			60	56.9	1.7	10.3	97.6	
18	Lena	15.6	5	12.5	1.7	61	24	4.2	12.2	146.9	11
19	Limpopo	20.7	80	78	10	42.1	44.1	35.4	4.7	205.6	26
20	Mackenzie	37	23.7	58.4	4.6	157	59.6	40	65.3	318.9	8.8
21	Magdalena	142.1	920	434.6	59	451	164	461	180.6	972.7	253

22(a)	Mekong (Chiang Saen)	75.1	336.0	154.1	20	284	106	78.7	71.6	715.4	78.2
22(b)	Mekong (Pakse)	74.1	313.2	126.2	22	289	89.4	73.9	72.1	680.7	103
22(c)	Mekong	199.8	200	503.7	37	761	279	340	260.6	1751	127
23	Mississippi	39.3	120	87	13	155	66.7	53.5	48.4	346.4	23
24	Murray Darling	16.3	29	158.2	5.5	18.9	25.2	176	14.3	55.5	3
25	Niger	6.5	33	8.6	3.1	15.3	8.6	2.9	0.6	60.8	26
26	Nile	26.4	40	153.6	14	52.6	39.1	85.4	36.8	193.8	14
27	Ob	20	6.3	36.4	4.1	73.7	33.3	29.5	14.1	202.7	6.8
28	Orange		100							27	
29	Orinoco	21.3	150	48.2	17	55.3	16.2	198	23.7	87	40
30	Parana	20.2	32.6	54.2	22	40.6	20.2	43.4	4.9	161.8	67
31	Red	176.3	1083.4	55.4	25	863	155	17.4	32.8	2017	173
32	Salween	198.7		282.4	17	747	427	371	6.5	2256	
33	Sao Francisco		9							320	
34	Senegal		8							7	
35	Severnaia Dvina		13							798	
36	Xijiang	120.9	149	169.2	31	517	177	45.1	81.7	1328	75
37	St. Lawrence	69.5	5	98.9	15	310	102	88.6	60.4	685.2	17
38	Tigris-Euphrates	46.4	57	46.9	6.2	196	63.9	97.8	40.5	422.9	36
39	Yana	3.6	14			19	16.4		0.9	32.6	
40	Yangtze	105.4	271.4	169.6	31	421	181	84	92.4	1101	54
41	Yellow	21.6	1183.6	97.1	18	49.1	48	79.1	44.0	141.2	
42	Yukon	39.7	62	24.5	6.7	172	63.9	6.7	50.5	387.2	28
43	Zaire	11.9	11.5	31.4	12	39.7	25.2	27.4	15.0	41.8	58
44	Zambesi	6.7	35	19.6	4.1	22	14.1	15.5	2.6	43.7	23
World mean		24.4	85.1	57.1	8.4	84.4	35.3	41.3	13.9	215.3	43.7

Specific flux (areal yield) is annual flux normalized to drainage area

Table S5. Statistically historical trends as year for concentrations and fluxes of TSS and major ions in the Mekong River.

(a) Chiang Saen

	Y	R^2	Adjusted R^2	p
Chiang Saen				
Observed mean (Y≥1985)				
TSS	34982.594 - 17.350 * Y	0.62	0.604	**<0.001**
Ca	-23.085 + 0.0254 * Y	0.0117	0	0.591
Mg	-160.989 + 0.0841 * Y	0.334	0.307	**0.002**
Na	-0.478 + 0.00523 * Y	0.003	0	0.797
K	7.976 - 0.00306 * Y	0.009	0	0.647
HCO_3	-267.252 + 0.188 * Y	0.04	0.004	0.304
Cl	-114.647 + 0.0621 * Y	0.14	0.11	**0.055**
SO_4	-637.008 + 0.329 * Y	0.241	0.211	**0.009**
Si	-103.120 + 0.0545 * Y	0.398	0.365	**0.003**
Discharge-weighted mean *(Y≥1985)*				
TSS	141012.367 - 70.494 * Y	0.416	0.343	**0.044**
Ca	455.992 - 0.216 * Y	0.153	0.0476	0.263
Mg	-140.933 + 0.0738 * Y	0.140	0.0321	0.287
Na	-79.510 + 0.0441 * Y	0.0708	0	0.457
K	3.817 - 0.00104 * Y	0.0006	0	0.945
HCO_3	-631.971 + 0.368 * Y	0.0592	0	0.498
Cl	37.533 - 0.0154 * Y	0.002	0	0.896
SO_4	768.743 - 0.378 * Y	0.231	0.134	0.16
Si	-16.117 + 0.0106 * Y	0.0105	0	0.778
Historical discharge-weighted mean *(Y≥1960)*				
TSS	6094.421 - 2.710 * Y	0.033	0.0119	0.217
Ca	9.450 + 0.00799 * Y	0.0349	0.0139	0.203
Mg	-0.150 + 0.00292 * Y	0.0335	0.0125	0.213
Na	-4.762 + 0.00640 * Y	0.0317	0.0106	0.226
K	1.642 + 0.0000432 * Y	0.0242	0.003	0.291
HCO_3	27.759 + 0.0350 * Y	0.0343	0.0133	0.208
Cl	-5.669 + 0.00602 * Y	0.0310	0.0099	0.232
SO_4	-5.961 + 0.0107 * Y	0.0328	0.0118	0.218
Si	2.239 + 0.00133 * Y	0.0349	0.0139	0.204
Annual flux *(Model 2; Y≥1960)*				
TSS	835.551 - 0.389 * Y	0.0347	0.0137	0.205
Ca	8.842 - 0.00335 * Y	0.0264	0.00523	0.270
Mg	1.764 - 0.000638 * Y	0.0268	0.0056	0.267
Na	2.049 - 0.000687 * Y	0.0261	0.005	0.272
K	0.638 - 0.000247 * Y	0.027	0.0058	0.265
HCO_3	32.859 - 0.0123 * Y	0.0264	0.0053	0.27
Cl	1.189 - 0.000332 * Y	0.0166	0	0.382
SO_4	4.871 - 0.00175 * Y	0.0266	0.0054	0.268
Si	1.651 - 0.000622 * Y	0.0267	0.0056	0.267

(b) Pakse

	Y	R^2	Adjusted R^2	p
Pakse				
Observed mean (Y≥1985)				
TSS	18360.047 - 9.101 * Y	0.421	0.397	**<0.001**
Ca	91.467 - 0.0343 * Y	0.0160	0	0.53
Mg	-65.449 + 0.0351 * Y	0.120	0.085	0.076
Na	-26.855 + 0.0167 * Y	0.00517	0	0.727
K	13.237 - 0.00586 * Y	0.0092	0	0.642
HCO_3	202.581 - 0.0600 * Y	0.006	0	0.701
Cl	-214.332 + 0.111 * Y	0.196	0.164	**0.021**
SO_4	-137.758 + 0.0768 * Y	0.0205	0	0.476
Si	-130.859 + 0.0685 * Y	0.206	0.159	**0.051**
Observed mean in the recent decade (2001-2011)				
Ca	1192.518 - 0.583 *Y	0.490	0.433	**0.016**
Na	- 1068 + 0.536*Y	0.638	0.602	**0.002**
HCO3	2679.242 - 1.294*Y	0.583	0.536	**0.006**
Cl	-760.724 + 0.383 *Y	0.284	0.205	**0.091**
Discharge-weighted observed mean *(Y≥1985)*				
TSS	28580.072 - 14.111 * Y	0.0872	0	**0.407**
Ca	-202.987 + 0.112 * Y	0.138	0.0519	0.234
Mg	-167.398 + 0.0860 * Y	0.142	0.0558	0.228
Na	303.926 - 0.150 * Y	0.262	0.18	0.107
K	39.003 - 0.0189 * Y	0.0187	0	0.689
HCO_3	-458.762 + 0.268 * Y	0.0425	0	0.520
Cl	-389.942 + 0.199 * Y	0.336	0.269	**0.048**
SO_4	-1003.664 + 0.511 * Y	0.395	0.334	**0.029**
Si	-331.721 + 0.169 * Y	0.451	0.39	**0.024**
Historical discharge-weighted mean *(Y≥1923) (modelled)*				
TSS	2582.962 - 1.051 * Y	0.0432	0.0315	0.058
Ca	9.811 + 0.00505 * Y	0.0720	0.0607	**0.014**
Mg	0.928 + 0.00140 * Y	0.0714	0.0601	**0.014**
Na	-0.521 + 0.00280 * Y	0.0749	0.0637	**0.012**
K	1.345 + 0.0000580 * Y	0.0740	0.0627	**0.012**
HCO_3	36.845 + 0.0174 * Y	0.0717	0.0603	**0.014**
Cl	-2.799 + 0.00372 * Y	0.0727	0.0614	**0.013**
SO_4	0.696 + 0.00569 * Y	0.0727	0.0614	**0.013**
Si	3.055 + 0.000948 * Y	0.0732	0.0619	**0.013**
Annual flux *(Model 2; Y≥1923)*				
TSS	1131.257 - 0.489 * Y	0.0391	0.0274	0.071
Ca	20.483 - 0.00715 * Y	0.0490	0.0374	**0.043**
Mg	3.690 - 0.00127 * Y	0.0491	0.0375	**0.043**
Na	4.750 - 0.00155 * Y	0.0491	0.0375	**0.043**
K	1.570 - 0.000562 * Y	0.0483	0.0367	**0.045**
HCO_3	75.189 - 0.0264 * Y	0.0490	0.037	**0.043**
Cl	4.631 - 0.00147 * Y	0.0491	0.0375	**0.043**
SO_4	10.049 - 0.00320 * Y	0.0443	0.0326	0.055
Si	5.571 - 0.00198 * Y	0.0489	0.0373	**0.043**

References

Amiotte Suchet P, Probst JL, Ludwig W. Worldwide distribution of continental rock lithology: Implications for the atmospheric/soil CO2 uptake by continental weathering and alkalinity river transport to the oceans. Glob Biogeochem Cycles 2003;17:1038.

http://multimedia.wri.org/watersheds_2003/ (read on 20 April 2014)

Huh Y, Tsoi MY, Zaitsev A, Edmond JM. The fluvial geochemistry of the rivers of Eastern Siberia: I. Tributaries of the Lena River draining the sedimentary platform of the Siberian Craton. Geochim Cosmochim Acta 1998a;62:1657–76.

Gaillardet J, Duprè B, Louvat P, Allègre CJ. Global silicate weathering and CO2 consumption rates deduced from the chemistry of large rivers. Chem Geol 1999;159:3–30.

Galy A, France-Lanord C. Weathering processes in the Ganges–Brahmaputra basin and the riverine alkalinity budget. Chem Geol 1999;159:31–60.

Karim A, Veizer J.Weathering processes in the Indus River Basin: implications from riverine carbon, sulfur, oxygen, and strontium isotopes. Chem Geol 2000;170(1–4):153–77.

Ludwig W, Probst JL, Kempe S. Predicting the oceanic input of organic carbon by continental erosion. Global Biogeochem. Cycl. 1996; 10: 23–41.

Meybeck M. Les fleuves et le cycle ge'ochimique des elements. Paris: Univ. Pierre et Marie Curie; 1984 [The'se de Doctorat d'Etat].

Meybeck M. Global occurrence of major elements in rivers. In: Drever JI, editor. Treatise on geochemistry, vol. 5. New York: Elsevier; 2004. p. 202–33.

Moon S, Huh Y, Qin J, Pho NV. Chemical weathering in the Hong (Red) River basin: rates of silicate weathering and their controlling factors. Geochim Cosmochim Acta 2007;71:1411–30.

Nemeth A, Paolini J, Herrera R. Carbon transport in the Orinoco River: the preliminary results. In: Degens ET, editor. Transport of carbon and minerals in major world rivers. Hamburg, Germany: Univ. of Hamburg; 1982. p. 357–64.

Sarin MM, Krishnaswamy S, Dilli K, Somayajulu BLK, Moore WS. Major ion chemistry of Ganga–Brahmaputra river system: weathering processes and fluxes of the Bay of Bengal. Geochim Cosmochim Acta 1989;53:997–1009.

Stallard RF, Edmond JM. Geochemistry of the Amazon 2. The influence of geology and weathering environment on the dissolved load. J Geophys Res 1983;88(C14): 9671–88.

SCOPE 42 - Biogeochemistry of Major World Rivers (http://www.scopenvironment.org/downloadpubs/scope42/chapter04.html; read on 20 April 2014)

Varol M, Gokot B, Bekleyen B, Sen B. Geochemistry of the Tigris River basin, Turkey: spatial and seasonal variations of major ion compositions and their controlling factors. Quat Int 2013;304:22–32.

Zhang SR, Lu XX, Higgitt DL, Chen CTA, Sun HG, Han JT. Water chemistry of the Zhujiang (Pearl River): natural processes and anthropogenic influences. J Geophys Res 2007;112:F01011. http://dx.doi.org/10.1029/2006JF000493.
